# Deposition of Histone Variant H2A.Z within Gene Bodies Regulates Responsive Genes

**DOI:** 10.1371/journal.pgen.1002988

**Published:** 2012-10-11

**Authors:** Devin Coleman-Derr, Daniel Zilberman

**Affiliations:** Department of Plant and Microbial Biology, University of California Berkeley, Berkeley, California, United States of America; National Institute of Genetics, Japan

## Abstract

The regulation of eukaryotic chromatin relies on interactions between many epigenetic factors, including histone modifications, DNA methylation, and the incorporation of histone variants. H2A.Z, one of the most conserved but enigmatic histone variants that is enriched at the transcriptional start sites of genes, has been implicated in a variety of chromosomal processes. Recently, we reported a genome-wide anticorrelation between H2A.Z and DNA methylation, an epigenetic hallmark of heterochromatin that has also been found in the bodies of active genes in plants and animals. Here, we investigate the basis of this anticorrelation using a novel *h2a.z* loss-of-function line in *Arabidopsis thaliana.* Through genome-wide bisulfite sequencing, we demonstrate that loss of H2A.Z in Arabidopsis has only a minor effect on the level or profile of DNA methylation in genes, and we propose that the global anticorrelation between DNA methylation and H2A.Z is primarily caused by the exclusion of H2A.Z from methylated DNA. RNA sequencing and genomic mapping of H2A.Z show that H2A.Z enrichment across gene bodies, rather than at the TSS, is correlated with lower transcription levels and higher measures of gene responsiveness. Loss of H2A.Z causes misregulation of many genes that are disproportionately associated with response to environmental and developmental stimuli. We propose that H2A.Z deposition in gene bodies promotes variability in levels and patterns of gene expression, and that a major function of genic DNA methylation is to exclude H2A.Z from constitutively expressed genes.

## Author Summary

Eukaryotes package their DNA to fit within the nucleus using well-conserved proteins, called histones, that form the building blocks of nucleosomes, the fundamental units of chromatin. Histone variants are specialized versions of these proteins that change the chromatin landscape by altering the biochemical properties and interacting partners of the nucleosome. H2A.Z, a conserved eukaryotic histone variant, is preferentially enriched at the beginnings of genes, though the significance of this enrichment remains unknown. We and others have shown that H2A.Z is conspicuously absent from methylated DNA across the genome in plants and animals. Typically considered a mark of epigenetic silencing, DNA methylation has more recently been discovered in the bodies of many genes. Here, we present evidence that the genome-wide anticorrelation between DNA methylation and H2A.Z enrichment in *Arabidopsis* is the result of DNA methylation acting to prevent H2A.Z incorporation. We demonstrate that the presence of H2A.Z within gene bodies is correlated with lower transcription levels and higher variability in expression patterns across tissue types and environmental conditions, and we propose that a major function of gene-body DNA methylation is to exclude H2A.Z from the bodies of highly and constitutively expressed genes.

## Introduction

In addition to packaging the DNA to fit within the cell, histones function to control the structure and accessibility of the chromatin environment by altering the biochemical properties of the nucleosome or through the recruitment of distinct binding partners. These actions promote changes in transcription that regulate the proper timing of developmental decisions and appropriate responses to the external environment. One such method of histone-mediated control comes from the exchange of the canonical histones with non-allelic histone variants, which alter the fundamental structure and stability of the nucleosome [1]–[4].

H2A.Z is one of the most enigmatic of these histone variants, as well as the most well-conserved, with a single origin at the root of eukaryotic evolution [1]. H2A.Z has been implicated in a number of apparently disparate and even contrary chromosomal processes, including heterochromatic silencing, gene activation, transcriptional memory, cell-cycle progression and thermal-sensory response [5]–[9]. A common aspect of H2A.Z biology is its enrichment within the few nucleosomes surrounding transcription start sites (TSS), which has been demonstrated by genome-wide localization experiments in protozoa, fungi, animals, and plants [10]–[19].

The conserved H2A.Z distribution pattern at the TSS in many species has lead to considerable effort to understand the effect of H2A.Z on transcription. H2A.Z enrichment at promoters in yeast is simultaneously required for transcription and inversely correlated with transcription level [11], [20], [21]. Studies in animals have reported that H2A.Z exhibits a positive correlation with transcription [12], [22], [23], although some have found that this relationship is only true up to a point, after which the association becomes negative [15], [24]. In plants, the relationship between H2A.Z at the TSS and transcription appears to be roughly parabolic, with the highest and lowest expressed genes having the least H2A.Z enrichment [16].

In the yeasts *Saccharomyces cerevisiae* and *Schizosaccharomyces pombe*, H2A.Z regulates genes that respond to changes in the environment [21], [25], [26], and loss-of-function mutants fail to react appropriately to external cues [27], [28]. *Arabidopsis thaliana* plants lacking PIE1 (*AT3G12810*), the plant homolog of the SWR1 catalytic subunit of protein complexes responsible for the deposition of H2A.Z in yeast and mammals [29]–[36], exhibit misregulation of many genes involved in the innate immune response [37]. Recent work has shown that Arabidopsis plants with a mutated *ARP6*, which encodes a component of the PIE1 complex, inappropriately express temperature response genes, leading to the proposal that H2A.Z may act specifically as a thermosensor in plants [8].

The genomic distribution and biological functions of DNA methylation, another well-conserved feature of chromatin, are in many aspects strikingly different from those of H2A.Z. DNA methylation in the form of 5-methylcytosine is present in all vertebrates examined to date, as well as in many invertebrates, fungi, and plants [24], [38], [39]. The primary function of eukaryotic DNA methylation has long been considered to be the silencing of the sequences it decorates, particularly transposable elements [40], although the recent discovery of gene body methylation in plants and animals, the functional significance of which is still unknown, has complicated this view [24], [38], [41]–[45]. Whereas H2A.Z is enriched near the TSS of most genes, TSS-proximate DNA methylation is strongly associated with transcriptional repression in plants and vertebrates [46].

Recently, we reported a strong, genome-wide anticorrelation between H2A.Z and DNA methylation in Arabidopsis, including in bodies of active genes [16]. Results from similar studies in vertebrates suggest that this anticorrelation is a conserved feature of eukaryotes [24], [47], [48]. In Arabidopsis, we showed that changes in DNA methylation caused by a mutation in the DNA methyltransferase *MET1* induced reciprocal alterations in H2A.Z deposition, demonstrating that DNA methylation antagonizes H2A.Z recruitment [16]. We also used a null mutation in *PIE1* (*pie1–5*) to examine the effect of disrupted H2A.Z function on DNA methylation. By coupling methylated DNA immunoprecipitation (MeDIP) to microarray analysis, we found a low magnitude but genome-wide DNA methylation increase in genes that suggested a mutual antagonism between H2A.Z and DNA methylation [16].

There is now considerable evidence that the PIE1 complex deposits H2A.Z into chromatin in Arabidopsis, though whether it has H2A.Z-independent functions, as has been shown for other eukaryotic SWR1 homologs, remains unclear [29], [49], [50]. Other Arabidopsis chromatin remodelers are probably also able to deposit H2A.Z, as does the yeast INO80 complex [51], because H2A.Z is incorporated into nucleosomes at low levels in *pie1* and *swr1* mutants [29], [34], [52]. Given that the sets of genes that are misregulated in H2A.Z and SWR1-related mutants only partially overlap in both *S. cerevisiae* and Arabidopsis [29], [37], we sought to use an H2A.Z-deficient plant line, as opposed to SWR1-related mutants, for further analysis of H2A.Z function.

Here, we describe the characterization of an H2A.Z loss-of-function line in *Arabidopsis thaliana*. We find that loss of H2A.Z in Arabidopsis has little effect on the level or profile of DNA methylation in genes, and propose that the global anticorrelation between DNA methylation and H2A.Z is primarily caused by the exclusion of H2A.Z from methylated DNA. We show that the level of H2A.Z enrichment in gene bodies is generally correlated with gene responsiveness and that lack of H2A.Z causes misregulation of genes that respond to a variety of stimuli. We propose that H2A.Z deposition in gene bodies promotes gene responsiveness, but may destabilize constitutive expression, and that a major function of gene body DNA methylation is to exclude H2A.Z from constitutively expressed genes.

## Results

### Construction of a near-null Arabidopsis *h2a.z* mutant line

**Figure 1:**
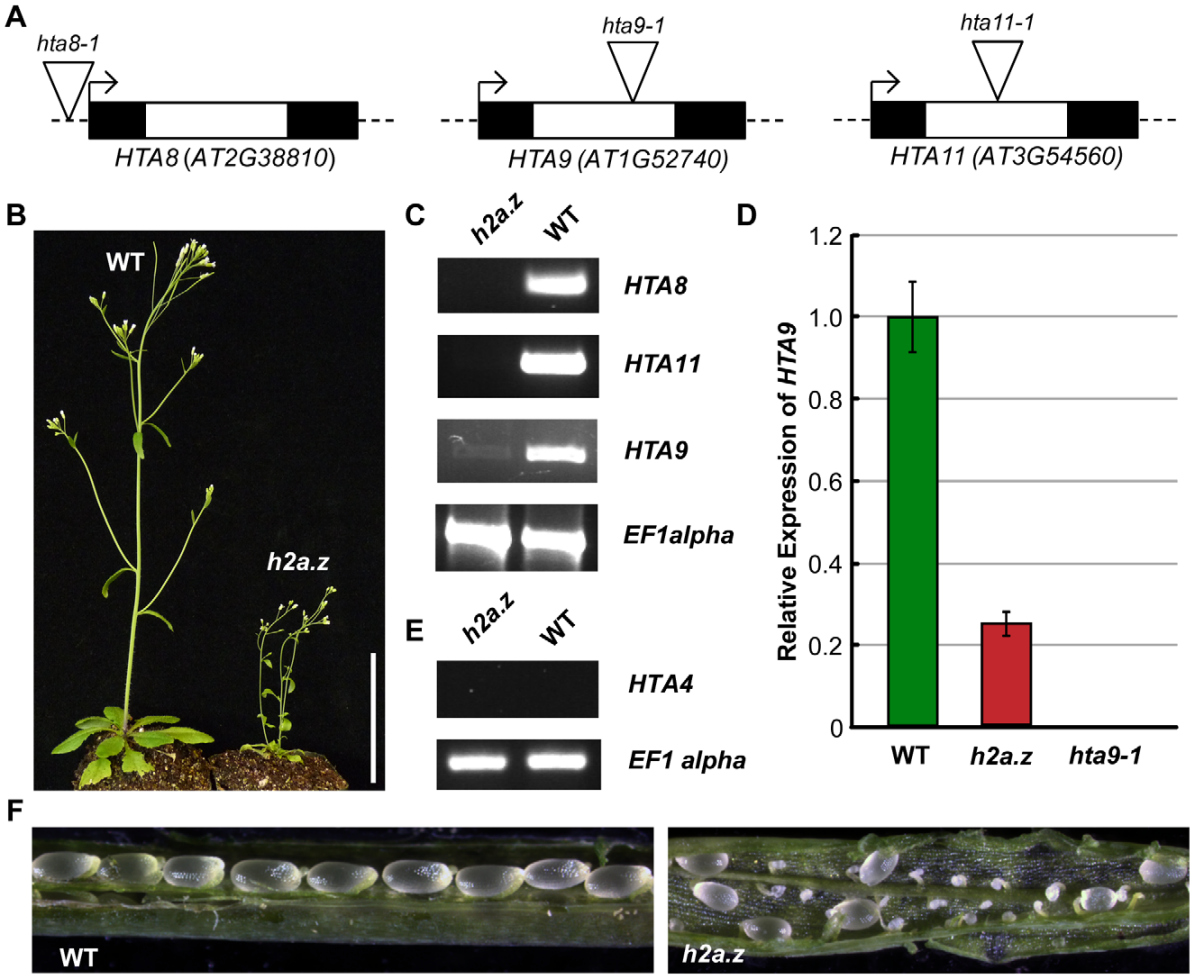
Construction of an *h2a.z* mutant line. (A) The three T-DNA insertions in *HTA8*, *HTA9*, and *HTA11*. Exons are represented by dark boxes, introns by unfilled boxes. Triangles represent the T-DNA insertion points. *hta9-1* and *hta11-1* are intronic insertions, while *hta8-1* is an insertion in the 5′ UTR. (B) WT and *h2a.z* plants at 21 days post germination. Scale bar = 3 cm. (C) RT-PCR of *HTA8*, *HTA9*, and *HTA11* in the *h2a.z* mutant line and WT control line. RT-PCR of *EF1alpha* was used as a loading control. (D) qPCR results for *HTA9* transcripts in the *h2a.z* and *hta9-1* mutants. Expression is normalized to WT levels; WT is shown in green, and *h2a.z* in red. Note that the level of *HTA9* in the *hta9-1* mutant is 0.040 percent of WT. (E) RT-PCR of *HTA4* in the *h2a.z* mutant line and a WT control. RT-PCR of *EF1alpha* was used as a loading control. (F) Ovule development in *h2a.z* mutant and WT siliques grown in LD conditions.

Three of the thirteen Arabidopsis *H2A* genes, *HTA8 (AT2G38810)*, *HTA9 (AT1G52740)*, and *HTA11 (AT3G54560)*, have been classified as encoding H2A.Z based on phylogenetic analyses [53], [54], and distribution patterns and genetic studies suggests that these proteins are largely functionally redundant [16], [33], [34]. Recently published work has demonstrated that a double mutant of *hta9-1* and *hta11-1* produced plants with phenotypes similar to those found in null *pie1–5* mutants [37]. To generate a line devoid of H2A.Z, we crossed *hta9-1* and *hta11-1* plants with a line bearing an insertion in *HTA8*, *hta8-1* (Figure 1A). Contrasting with recent evidence that individual knockouts of the two vertebrate H2A.Z isoforms exhibit different phenotypes [55], we did not observe morphological abnormalities in any of the three single mutant lines. The resulting triple mutant line, which we will refer to as *h2a.z,* is both viable and phenotypically distinguishable from WT (Figure 1B). Transcripts of *HTA8* and *HTA11* were not detectable in the *h2a.z* mutant by RT-PCR, but low levels of *HTA9* RNA were present (∼26% of wild-type; Figure 1C and 1D) in *h2a.z* plants but not in *hta9-1* single mutants, suggesting that the intronic T-DNA insertion in *HTA9* is spliced out in a fraction of transcripts, as confirmed by sequencing of the cDNA (Figure S1). To test whether this low level of expression was the result of a genetic rearrangement at the *HTA9* locus that occurred in our crosses, we recreated the *h2a.z* line using *hta9-1* plants lacking *HTA9* transcript (Figure 1D). The *h2a.z* progeny from the independent cross produced similar phenotypes to the original *h2a.z* line and similar RT-PCR results for *HTA9*, suggesting upregulation of *HTA9* in the triple mutant.

A fourth gene, *HTA4 (AT4G13570)*, is the closest H2A family member to the three *H2A.Z* genes and has been categorized as H2A.Z-like [54], but all publically available data indicate that *HTA4* is not expressed at significant levels in any WT tissue. To ensure that *HTA4* is not upregulated as a result of the drop in H2A.Z levels in our *h2a.z* line, we tested the expression of *HTA4* by RT-PCR (Figure 1E), and did not detect *HTA4* RNA in *h2a.z* or in WT. Taken together, our data indicate that the *h2a.z* line has less than ten percent of wild-type *H2A.Z* transcript levels. Despite reduced fertility (Figure 1F), *h2a.z* plants are viable and produce offspring, differing markedly from the lethality of strong H2A.Z mutations in other multicellular organisms [15], [56], [57], [58], [59].

### The *h2a.z* mutant phenotype is distinct from that caused by lack of PIE1

**Figure 2:**
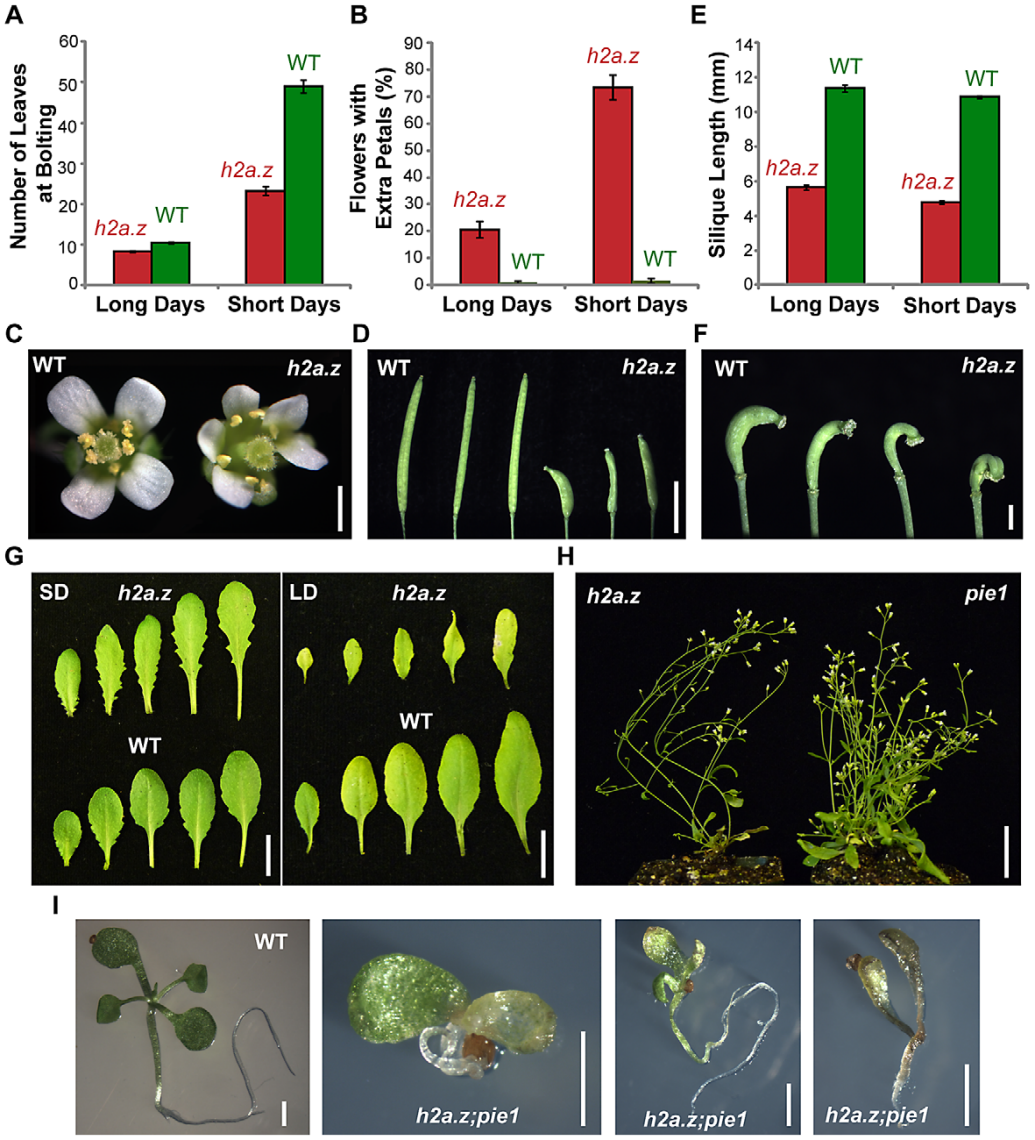
Characterization of the *h2a.z* mutant phenotype. (A) Average flowering time of the *h2a.z* mutant and WT control, as measured by the average number of rosette leaves at the time of bolting (cotyledons not included). Measurements were taken for 50 *h2a.z* and WT plants in both long day (LD, 16 h light/8 h dark) and short day (SD, 8 h light/16 h dark) conditions. (B) Average percentage of early flowers with extra petals, as measured in the first ten flowers of *h2a.z* mutant and WT plants. Measurements were averaged for 50 *h2a.z* and WT plants in both LD (16 h light/8 h dark) and SD (8 h light/16 h dark) conditions. (C) *h2a.z* mutant (right) and WT (left) flowers grown in SD. Scale bar = 2 mm. (D) *h2a.z* mutant (right) and WT (left) siliques. Scale bar = 4 mm. (E) Average mature silique length in *h2a.z* mutant and WT plants. Measurements were averaged for 10 mature siliques from each of 50 *h2a.z* and WT plants in both LD (16 h light/8 h dark) and SD (8 h light/16 h dark) conditions. (F) Strong curvature of developing *h2a.z* mutant siliques. Scale bar = 1 mm. (G) Rosette leaf morphology in *h2a.z* mutant and WT plants grown in SD (40 days; left panel) and in LD (28 days; right panel). Scale bar = 1 cm. (H) Phenotypes of *h2a.z* and *pie1–5* mutants grown in LD at 6 weeks post germination. Scale bar = 1 cm. (I) Phenotypes of WT and *h2a.z;pie1* double mutants at 14 days post germination. Scale bar = 1 mm.

We measured the number of leaves present when the plant produced its first flower buds in *h2a.z* and WT (Figure 2A). In short days (SD), the *h2a.z* line flowered significantly earlier than WT, with 23.2+/−1.1 leaves vs. 49.7+/−1.5 leaves (P-value<0.0001, two sample T-test). In long days (LD), the difference in flowering time between *h2a.z* and WT was less pronounced, with 8.3+/−0.2 leaves and 10.6+/−0.2 leaves, respectively (P-value<0.0001), but the difference in rosette size and plant stature was greater in LD than SD (Figure S2). Of the first ten flowers, 22+/−3.1% in LD and 76+/−4.6% in SD exhibited extra petals (between 5 and 8) in the *h2a.z* mutant line, compared to 1.5+/−0.6% (LD) and 2+/−0.8% (SD) in WT (Figure 2B and 2C). The *h2a.z* mutant also exhibited short, thickened siliques, a phenomenon potentially related to decreased fertility. The *h2a.z* siliques averaged 4.8+/−0.1 and 5.6+/−0.1 mm in length under LD and SD conditions, compared to 10.6+/−0.1 and 11+/−0.2 mm for WT (Figure 2D–2F). The *h2a.z* phenotypes described above, as well as increased leaf serration and petiole length in SD (Figure 2G), are similar to those previously published for *hta9-1*; *hta11-1* and *pie1–5* mutants [32], [37], [60].

The *h2a.z* line exhibited several phenotypes not previously reported for *pie1–5* or *hta9-1;hta11-1.* First, while both *pie1–5* and *h2a.z* have reduced stature, *pie1–5* plants are more severely dwarfed and have a bushy, extensively branched architecture, whereas *h2a.z* plants are spindly and have trouble remaining upright (Figure 2H). This aspect of the *h2a.z* phenotype might be caused by contributions from the WS ecotype of *hta8-1* (all other lines are in the Col ecotype), but this is unlikely because the WT siblings from the same cross do not show these traits. Additionally, many of the siliques in the *h2a.z* mutant exhibited a strong asymmetric curvature, most likely due to the improper fusion of its carpels (Figure 2F). Other novel phenotypes occurred only rarely, affecting multiple aerial plant tissues including leaf and stem structures, but were most prevalent among floral organs (Figure S3). The most striking examples were the inappropriate emergence of petals and stamens directly from the stem, and flowers with improperly fused carpels, leading to severely compromised reproductive structures.

A mutation in yeast *swr1* (*pie1*) ameliorates many of the phenotypes observed with the *htz1* (*h2a.z*) single mutant, as well as the severe phenotypes of the double mutant between *htz1* and *set3* [61], [62]. The cause of the *htz1* phenotypes was proposed to be chromatin disruption by the SWR1 complex in the absence of its proper substrate, a hypothesis supported by SWR1-dependent accumulation of DNA damage in the absence of *htz1*. To test whether simultaneous removal of the PIE1 chromatin remodeler and H2A.Z would reduce the severity of phenotypes seen in *h2a.z* plants, we crossed the *h2a.z* mutant line to *pie1–5*. Contrary to the results from yeast, the phenotype of the Arabidopsis double mutant is more severe than that of either parent – progeny exhibit early developmental arrest, dying shortly after germination (Figure 2I). Taken together with the phenotypic disparity, our results suggest that H2A.Z and PIE1 have non-redundant functions in Arabidopsis. Because *h2a.z* is not a complete loss of function line, the stronger phenotype of *h2a.z*; *pie1* plants might be caused by a further reduction of H2A.Z incorporation into chromatin, but nevertheless demonstrates that *pie1–5* does not entirely abolish H2A.Z function. While we cannot rule out the possibility that all *pie1* phenotypes are associated with H2A.Z, we consider this unlikely because of the stronger effect of *pie1* on plant architecture compared to *h2a.z* (Figure 2H).

### Lack of H2A.Z does not substantially perturb genic DNA methylation

**Figure 3:**
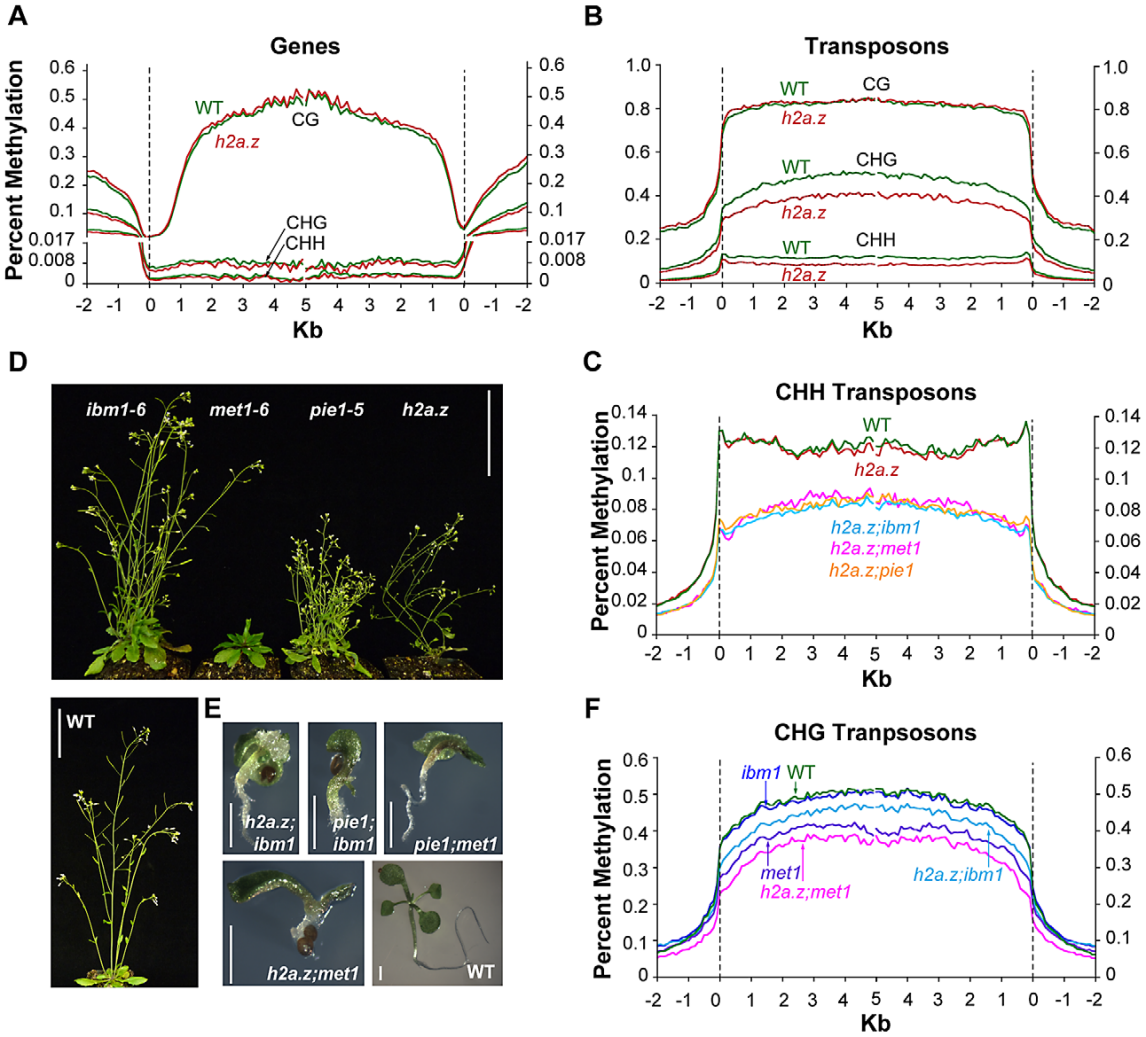
DNA methylation profiles of *h2a.z*-related mutants. (A) Profiles of CG, CHG, and CHH DNA methylation in two replicates each of cauline leaf *h2a.z* and WT. Genes were aligned at the 5′ end (left half of panel) and the 3′ end (right half of panel) and average methylation levels for each 100-bp interval are plotted from 2 kb away from the gene (negative numbers) to 5 kb into the gene (positive numbers). WT methylation is represented by the green traces, while *h2a.z* methylation is represented by red traces. The dashed lines at zero represents the point of alignment. The Y-axis was partitioned at 0.017 and the lower portion expanded to aid in the visibility of CHG and CHH traces. (B) Profiles of CG, CHG, and CHH DNA methylation in transposons in *h2a.z* and WT, aligned as genes were in (A). WT methylation is represented by the green traces, while *h2a.z* methylation is represented by red traces. (C) Profiles of average CHH DNA methylation in TEs. TEs were aligned and average methylation levels determined as in (B). WT methylation is represented by the green trace, *h2a.z* by the red trace, *h2a.z;met1* by the pink trace, *h2a.z;ibm1* by the light blue trace, and *h2a.z;pie1* by the orange trace. (D) Phenotypes of *ibm1–6*, *met1–6*, *pie1–5*, *h2a.z* and WT plants grown in LD conditions at 6 weeks post germination. Scale bars = 3 cm. (E) Phenotypes of *h2a.z*;*ibm1*, *h2a.z*;*met1*, *pie1*;*ibm1*, *pie1*;*met1* double mutants and WT seedling at 14 days post germination grown in LD conditions. Scale bars = 1 mm. (F) Profiles of average CHG DNA methylation in TEs, as in (C). WT methylation is represented by the green trace, *met1* by the purple trace, *h2a.z;met1* by the pink trace, and *h2a.z;ibm1* by the light blue trace, and *ibm1* by the dark blue trace.

To test our hypothesis that H2A.Z protects genes from DNA methylation, we generated genome-wide methylation profiles for the *h2a.z* mutant and WT using shotgun bisulfite sequencing. Because plants have DNA methylation in three different sequence contexts, CG, CHG, and CHH (H = A, T or C), which are largely controlled by distinct families of methyltransferases and have different genome-wide distributions [24], [38], it is advantageous to use an assay that has single base-resolution to distinguish between these contexts. Two biological replicates each of *h2a.z* and WT were generated for each of three different tissue types that represent different stages along a developmental continuum: 14 day-old whole seedlings, 6 week-old rosette leaves, and 6 week-old cauline leaves. One biological replicate was taken from the original *h2a.z* mutant line, and the second from the additional *h2a.z* line generated from independent crosses with the same T-DNA insertional alleles. Analysis of the average methylation levels across all genes revealed that a loss of H2A.Z in Arabidopsis has little effect on the global patterns of DNA methylation in CG, CHG or CHH contexts (Figure 3A and Figure S4). For comparison, we generated bisulfite sequencing data for two biological replicates each of *pie1* and sibling WT seedlings, and one replicate of *h2a.z;pie1* seedlings. As with the results for the *h2a.z* mutant, the *pie1* and *h2a.z;pie1* mutants showed only subtle changes compared with WT in the global patterns of genic DNA methylation (Figure S5). Previously, we used locus-specific bisulfite sequencing to validate our microarray results at five candidate genes scored as hypermethylated in the *pie1* mutant; all five showed modest but consistent gains in CG methylation [16]. Similar analyses performed here on both our *h2a.z* and *pie1* mutants demonstrate an overall consistency between current and previous *pie1* data. They also show that the hypermethylation at these loci in *pie1* is less consistently present in *h2a.z* (Figure S6). Statistical analyses of the methylation differences between the *h2a.z*, *pie1*, and *h2a.z;pie1* mutants and their respective WT controls suggest that there are subtle increases in genic methylation as a result of H2A.Z loss (Figure S7). These results are consistent with our published data, showing a small but statistically significant increase of genic methylation in the *pie1* mutant [16]. However, the quantitative data generated here demonstrate that this increase is of a very low magnitude and is unlikely to substantially contribute to the global anticorrelation between H2A.Z and DNA methylation.

Unexpectedly, the *h2a.z* mutant exhibited tissue-specific DNA methylation changes in transposable elements (TEs; Figure 3B and Figure S8). CG methylation was marginally increased over wild-type in four of the six replicates, with the most consistent change in seedlings, whereas CHG methylation decreased more heavily in the older tissues, though there is considerable variation between replicates (Figure 3B and Figure S8). CHH methylation was substantially reduced specifically in cauline leaves (Figure 3B and Figure S8). Kernel density estimations of these changes demonstrate that the majority of transposons show a modest change in methylation, rather than a larger effect in a small subset of TEs (Figure S9). Analyses of DNA methylation in *pie1* and *h2a.z;pie1* seedlings show that, like *h2a.z* seedlings, these lines exhibit increased CG methylation in TEs (Figure S10). Curiously, the *h2a.z;pie1* seedlings exhibit decreases in CHG and CHH TE methylation that are not seen in seedlings of *pie1* or *h2a.z*, but which are reminiscent of the decreases in *h2a.z* plants later during development (cauline and rosette leaves; Figure 3B–3C and Figure S10). Our data indicate that whereas a loss of H2A.Z does not substantially change DNA methylation within genes, lack of H2A.Z affects TE methylation in all three sequence contexts in a development-specific manner.

We hypothesized that a larger effect of H2A.Z on DNA methylation may be undetectable in our *h2a.z* mutant due to the carefully targeted nature of routine DNA methylation maintenance. By this logic, H2A.Z's role may be to protect against random and spurious accumulation of DNA methylation at the TSS over evolutionary timescales, rather than to act as a barrier to regular DNA methylation processes. To test this hypothesis, we performed crosses of *h2a.z* and *pie1* to two methylation mutants, *ibm1–6* and *met1–6,* in which normal methylation targeting to genes is perturbed. We expected that if H2A.Z were acting to prevent methylation from accumulating at the TSS, there would be greater increases in methylation in these double mutants than in the parental lines. *IBM1* (*AT3G07610*) encodes a H3 lysine 9 demethylase, *MET1* (*AT5G49160*) encodes the primary CG DNA methyltransferase, and both *ibm1* and *met1* mutations cause increased CHG methylation in gene bodies [43], [44], [63], [64], [65], [66], [67]. Single mutant plants are viable and fertile (Figure 3D), but *h2a.z*;*ibm1*, *h2a.z*;*met1*, *pie1;ibm1*, and *pie1;met1* double mutants die shortly after germination and exhibit severe developmental abnormalities, including the production of undifferentiated callus-like material, under-sized root systems, and premature flowering (Figure 3E).

Bisulfite sequencing of *h2a.z;ibm1*, *h2a.z*;*met1,* and *pie1;ibm1* seedlings revealed that a loss of H2A.Z does not strongly alter the genic methylation profile in any context from that seen in the parental backgrounds (Figures S11, S12, S13). Statistical analyses of the CG methylation differences between the *h2a.z;ibm1*, *pie1;ibm1*, and the *ibm1* control suggest that the loss of H2A.Z in these double mutant lines leads to a subtle increase in genic methylation, as we found in the *h2a.z* and *pie1* single mutants (Figure S14). Once again, however, the magnitude of this change is extremely small.

The defining characteristic of *ibm1* mutants is a major increase in genic CHG methylation [63], [64], [68]. The *h2a.z;ibm1* and *pie1;ibm1* double mutant lines were generated such that *h2a.z;ibm1* seedlings were newly homozygous for *ibm1* (first generation), whereas *pie1;ibm1* seedlings came from first generation *ibm1* homozygous parents (second generation). The *h2a.z;ibm1* seedlings in their first generation of *ibm1* homozygosity have higher levels of CHG methylation than first generation *ibm1* seedlings, and *pie1;ibm1* seedlings in their second generation of *ibm1* homozygosity have lower levels of CHG methylation than second generation *ibm1* seedlings (Figure S12). Both first generation datasets, *h2a.z;ibm1* and *ibm1,* show similar levels of CHH hypermethylation to one another; likewise, the second generation *pie1;ibm1* and *ibm1* data exhibit similar CHH hypermethylation levels (Figure S13). Importantly, the control data show that genic CHG and CHH methylation is unstable in ibm1, increasing greatly in the second generation (Figure S12), making interpretation of changes in *h2a.z;ibm1* and *pie1;ibm1* CHG methylation difficult.

Whereas there is little difference between the double mutant lines and their parental lines in TE CG methylation (Figure S15), we found CHG hypomethylation in the double mutants as compared to their respective parental lines (Figure 3F and Figure S16). Additionally, while CHH methylation is unaltered in *h2a.z* and *pie1* seedlings, there is a significant reduction of TE CHH methylation in *h2a.z*;*ibm1*, *h2a.z*;*met1,* and *pie1;ibm1* seedlings compared to the *ibm1* and *met1* single mutants, which is similar to the reduction seen in *h2a.z;pie1* seedlings (Figure 3C and Figure S17). Taken together, our results suggest that while H2A.Z may play a modest role in the regulation of DNA methylation in TEs, the genome-wide anticorrelation between H2A.Z and DNA methylation is due to DNA methylation preventing the incorporation of H2A.Z.

### H2A.Z is enriched in responsive genes

**Figure 4:**
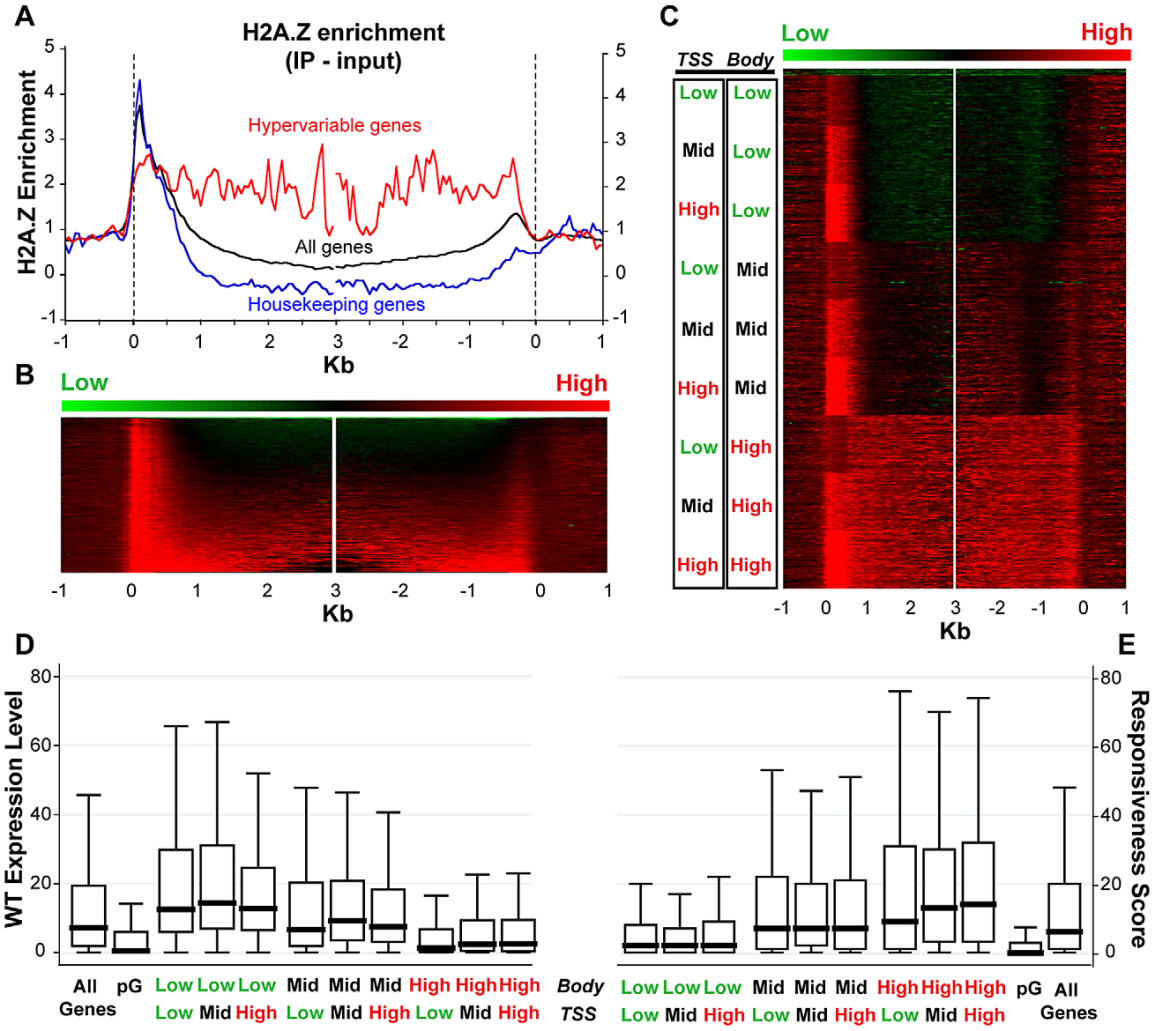
H2A.Z enrichment in gene bodies is associated with lower expression and higher responsiveness. (A) Average profile of H2A.Z enrichment (IP - input) across gene bodies for all genes (black trace, n = 21,675), housekeeping genes (blue trace, n = 371) and hypervariable genes (red trace, n = 117), as defined in Aceituno et. al. 2009 [70]. Genes were aligned at the 5′ end (left half of panel) and the 3′ end (right half of panel) and average H2A.Z enrichment levels for each 50-bp interval are plotted from 1 kb away from the gene (negative numbers) to 3 kb into the gene (positive numbers). To avoid averaging H2A.Z enrichment at the 5′ and 3′ ends of short genes into the H2A.Z body distribution, we do not use data within 1 kb of the opposite end of the gene for “all” and “housekeeping” genes. For “hypervariable” genes, we do not use data within 500 bp of the opposite end of the gene – analysis excluding 1 kb produces a very similar but much noisier trace due to fewer data points. (B) A heat map of H2A.Z enrichment (IP - input) for the genes represented by the “all” black trace in panel (A), aligned exactly as in panel (A). Genes were sorted (top to bottom) from lowest to highest H2A.Z enrichment across their bodies (from 500 bp after the TSS to 500 bp before the 3′ end). (C) The H2A.Z enrichment data were clustered into nine approximately equal sized groups based on three tiers (low, mid, high) of H2A.Z enrichment at the TSS (0 to 500 bp from the TSS) and across the body (from 1 kb after the TSS to 1 kb before the 3′ end to avoid contamination from the 5′ and 3′ H2A.Z peaks; genes under 2.1 kb were discarded); 158 pseudogenes, with very low levels of H2A.Z enrichment, were removed from the category representing lowest enrichment and clustered together at the top of the heat map. 12,237 genes are shown. (D) and (E) Box plots of WT expression levels (D) and responsiveness scores (E) for all genes partitioned into the nine H2A.Z-enrichment clusters in (C), as well as for all genes and for 158 pseudogenes (pG). Each box represents the middle 50% of the distribution, with the horizontal black lines marking the medians. The lines extending vertically from each box represent values that fall within 1.5 times the height of the box.

Given the published work linking H2A.Z with regulation of several types of genes that respond to the environment [8], [21], [26], [27], [28], [37], [69], we sought to examine H2A.Z enrichment with respect to gene responsiveness. To do so, we generated a genome-wide map of H2A.Z using our published tagged H2A.Z Arabidopsis line [16] by coupling affinity purification of H2A.Z-bound DNA with high-throughput sequencing. Metaanalyses of the new dataset demonstrate a strong peak of H2A.Z at the 5′ end and a smaller peak at the 3′ end of most genes, with varying levels of H2A.Z distributed within gene bodies (Figure 4A–4C). Our new data are consistent with our published microarray results, though the resolution provided by high-throughput sequencing is significantly better (Figure S18). To determine if a loss of H2A.Z had a preferential effect on genic methylation in the subset of genes that were enriched for H2A.Z in WT, as suggested by our published results in *pie1*, we compared the *h2a.z* and WT bisulfite sequencing datasets for those genes with the most and least H2A.Z across the TSS and the gene-body (Figure S19). Although there are major methylation differences between these four groups of genes, the profiles of *h2a.z* and WT within each group were virtually indistinguishable from one another. However, the subtle increase in genic methylation we detected in *h2a.z*, *pie1*, and *h2a.z;pie1* plants was stronger in H2A.Z enriched genes (Figure S7), consistent with our published results [16].

In support of earlier data [16], [24], we found a negative correlation between H2A.Z enrichment in gene bodies and WT transcript levels (Spearman's rho = −0.4039, P-value<0.0001). Genes with the most gene body H2A.Z (n = 4,081, Table S1) have median WT expression more than six-fold lower than that of genes with the lowest H2A.Z within their bodies (n = 3,920, Table S2) (Figure 4D). In comparison, levels of H2A.Z enrichment near the TSS showed a different trend: genes with the most and least H2A.Z at the TSS had lower levels of expression than those with intermediate levels of H2A.Z (Figure 4D), as we showed earlier for both Arabidopsis and pufferfish [16], [24].

We also discovered a positive correlation between enrichment of H2A.Z across gene bodies and gene responsiveness – the degree to which a gene is differentially expressed among different tissue types or experimental conditions (including hormone, nutrient, and chemical treatments, as well as biotic or abiotic stimulus), with higher response scores associated with greater differential expression [70]. H2A.Z body-enriched genes (n = 4,081) have a six-fold higher median gene responsiveness score than that of genes with the lowest H2A.Z levels across their bodies (n = 3,920) (Figure 4E). Levels of H2A.Z at the TSS are considerably less correlated with response score than levels of H2A.Z in the body (Spearman's rho = 0.0748 and 0.3325, P-values<0.0001, respectively), and highly responsive genes have more body H2A.Z than genes with low responsiveness (Figure S20). The least and most responsive genes [70], defined as housekeeping genes (n = 371) and hypervariable genes (n = 117) [70], are depleted for and enriched in H2A.Z across the gene body, respectively (Figure 4A and Figure S20). These results suggest that H2A.Z deposition in the gene body may facilitate rapid activation or inactivation of genes.

### H2A.Z regulates responsive genes

**Figure 5:**
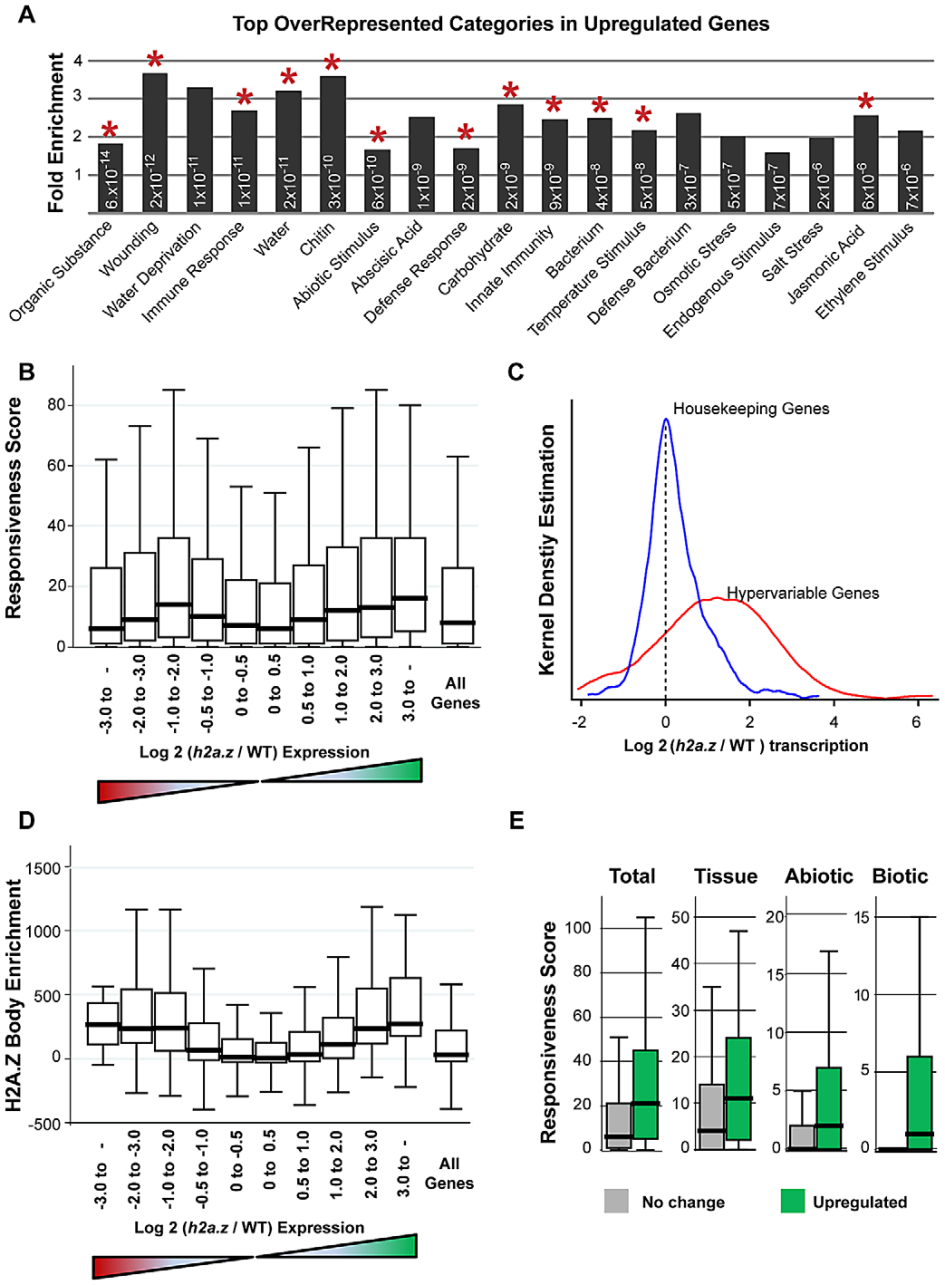
Genes with H2A.Z gene body enrichment are misregulated in *h2a.z*. (A) Fold enrichment of the top 19 over-represented GO categories in the 1,800 upregulated genes in the *h2a.z* mutant, all of which are response-related. For simplicity, all categories have had “Response to” removed from their names, with the exception of “Immune response” and “Defense response”. All categories have P-values for over-representation less than 1×10^−5^, and each P-value is indicated within the respective bar. GO terms that also appear as overrepresented in similar analyses done with genes upregulated in at least two of the three *h2a.*z, *pie1*, and *hta9;hta11* mutant datasets are marked with a red asterisk. (B) Box plots of Responsiveness Score for all genes partitioned by the degree of up and downregulation in the *h2a.z* mutant. Genes are grouped into bins based on increasing log_2_ (*h2a.z*/WT) expression level, ranging from −8.6 to 9.8; a separate bin shows the Responsiveness Score for all genes. The red and green triangles below the X-axis respectively represent decreased and increased expression in the mutant over WT. (C) Kernel density plots for transcriptional changes in the *h2a.z* mutant (log_2_ (*h2a.z*/WT) transcription) for housekeeping genes (blue, n = 384) and hypervariable genes (red, n = 123). (D) Box plots of H2A.Z body-enrichment (IP - input) for all genes with H2A.Z body enrichment scores (n = 12,237) partitioned by degree of up and downregulation. Genes are grouped as in (B). (E) Box plots for responsiveness, broken down by subcategory, in the 2-fold upregulated genes (n = 1,800, shown in green) and the least misregulated genes (less than 1.4-fold up or down regulated, n = 9,300, shown in grey and labeled “No change”). Subcategories and associated responsiveness scores are from [70], and represent the three major types of stimuli: developmental (tissue), abiotic, and biotic.

To determine which genes are misregulated upon loss of H2A.Z, we profiled the transcriptomes of the *h2a.z* mutant and WT in 4-week old rosette leaves with three replicates each of RNA sequencing. 1,800 genes were upregulated and 544 genes were downregulated in *h2a.z* with a P-value cut-off of 0.001. This is consistent with transcriptome analyses of *hta9;hta11* and *pie1,* which showed three-fold and two-fold more genes upregulated than downregulated, respectively [37]. The genes exhibiting up and downregulation in the *h2a.z* mutant show statistically significant overlap with lists of up and downregulated genes in *pie1* and *hta9;hta11* [37], despite differences in tissues type, developmental stage, growth conditions, and transcriptional profiling platform used to generate these data (Figure S21). Gene Ontology analysis of the misregulated genes in *h2a.z* revealed enrichment of categories related to immune response (P-value = 8.6×10^−9^) and temperature response (P-value = 4.8×10^−8^), consistent with previous studies of *pie1* and *arp4* mutants [8], [37] (Table S3). Strikingly, all of the most-enriched categories (P-value<1×10^−5^) are specifically response-related, and include many previously unreported GO-terms involved in the perception of a variety of external cues (Figure 5A). Many of these GO terms are also overrepresented in the smaller subset of genes upregulated in at least two of the *h2a.z*, *pie1*, and *hta9;hta11* mutant datasets (Figure 5A).

Consistent with our Gene Ontology analysis (Tables S3, S4), we discovered a relationship between the degree of misregulation in the *h2a.z* mutant and the responsiveness score of a gene (Figure 5B). Genes exhibiting greater than 4-fold upregulation (n = 938) had a 2.5-fold higher median responsiveness score than that of the least upregulated genes (less than 1.4-fold up or downregulated, n = 9,300). The relationship between downregulation and response score, on the other hand, was roughly parabolic, with the most downregulated and least downregulated genes showing the lowest levels of responsiveness, and genes with intermediate levels of downregulation (2 to 4-fold) showing the greatest responsiveness (Figure 5B). Hypervariable genes are generally strongly upregulated in *h2a.z* plants, despite a lack of change in DNA methylation (Figure S20), whereas the expression of housekeeping genes is largely unchanged (Figure 5C).

Because H2A.Z is enriched in bodies of response genes, we investigated whether changes in transcriptional regulation in the *h2a.z* mutant correlated with specific H2A.Z enrichment patterns in WT. As expected, we found a positive relationship between misregulation in the *h2a.z* line and H2A.Z gene body enrichment (Figure 5D) (Spearman's rho = 0.2634 for downregulated genes and 0.2540 for upregulated genes, P-value<0.0001). Genes with the greatest misregulation (greater than four-fold up or downregulated, n = 1,258) have more than a 36-fold higher median H2A.Z-body enrichment score than that of genes with the lowest levels of change in transcription between *h2a.z* and WT (less than 1.4-fold up or downregulated, n = 9,300). Taken together, our data demonstrate that loss of H2A.Z leads to a general transcriptional misregulation of response genes that are enriched for H2A.Z within the gene body in wild type, including genes that respond to developmental, biotic, and abiotic stimuli (Figure 5E). Our results also suggest that one function of gene body methylation, which is strongly anticorrelated with gene responsiveness in plants and animals [70], [71], [72], is the exclusion of H2A.Z from the bodies of constitutively expressed genes.

## Discussion

We have generated a viable H2A.Z-deficient mutant line in *Arabidopsis thaliana* that shares many, but not all of the phenotypic characteristics of *pie1* mutants. We show that unlike in yeast, combining Arabidopsis *h2a.z* and *pie1* mutations exacerbates the phenotype. Loss of H2A.Z does not substantially affect the level or profile of DNA methylation in genes, even when combined with mutations that alter the normal genic methylation landscape, whereas DNA methylation in transposons is perturbed in a tissue-dependent manner. We show that differences in gene body H2A.Z levels are correlated with gene expression and gene responsiveness. Finally, we show that a loss of H2A.Z causes misregulation of many genes involved in the response to developmental and environmental cues, and that these genes tend to have high levels of gene-body H2A.Z.

### Residual H2A.Z function remains in *pie1* mutant plants

Whereas the fungi *S. pombe* and *S. cerevisiae* can tolerate mutations in H2A.Z [27], [73], H2A.Z is essential in many species, including *Tetrahymena thermophila*, *Drosophila melanogastor*, *Xenopus laevis*, *Caenorhabditis elegans* and mice [15], [56], [58], [74], [75]. Consequently, many studies of H2A.Z function outside of yeast have utilized mutants in components of the chromatin remodelers that deposit H2A.Z to emulate H2A.Z loss-of-function [8], [34], [37], [69]. The substantial overlap between the phenotypes of Arabidopsis *pie1* and *h2a.z* mutants suggests that PIE1 is the primary remodeler responsible for H2A.Z deposition. However, *h2a.z;pie1* double mutants exhibit early developmental arrest not seen in either of the single mutant lines, indicating that H2A.Z may be deposited in the absence of the PIE1 complex, potentially by the Arabidopsis homolog of INO80 [76], which can deposit H2A.Z in yeast [51]. The PIE1 complex might also have H2A.Z-indpendent roles, as has been hypothesized for the PIE1/SWR1 orthologs in animals [49], [50]. Indeed, a recent study showed that H2A.Z deposition by p400 and SRCAP, the human orthologs of SWR1, could not account for all the regulatory roles of these complexes [50]. These results emphasize that phenotypes caused by mutations in chromatin remodeling complexes must be interpreted with caution.

### DNA methylation excludes H2A.Z from chromatin

**Figure 6:**
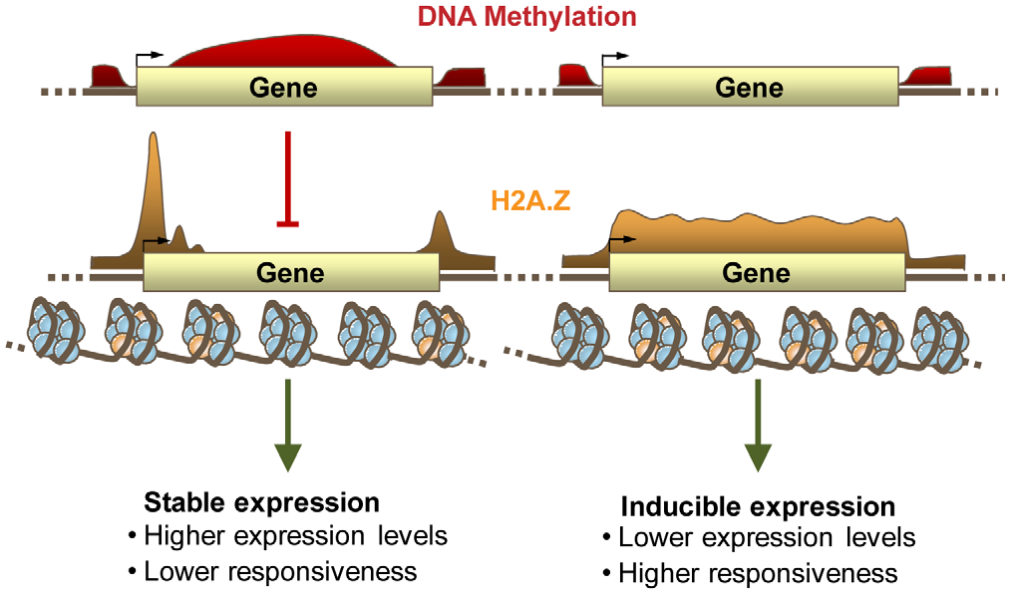
DNA Methylation, H2A.Z, and expression patterning. A proposed model for the relationship between DNA methylation (shown in red) and H2A.Z (shown in yellow) within genes, and their relationship with gene responsiveness and transcript level. Gene body methylation prevents the incorporation of H2A.Z within the bodies of highly and constitutively expressed genes. H2A.Z within unmethylated gene bodies regulates the expression of inducible genes.

DNA methylation and H2A.Z are tightly anticorrelated in plants and animals [16], [24], [47], [48], and we have shown that DNA methylation quantitatively excludes H2A.Z from chromatin [16]. Here, we demonstrate that H2A.Z does not have a large influence on DNA methylation in genes, even when genic DNA methylation is in flux, but that loss of H2A.Z does cause a small increase in genic methylation, particularly in H2A.Z-enriched genes, consistent with our earlier results [16]. The magnitude of these changes is unlikely to substantially contribute to the genome-wide anticorrelation between DNA methylation and H2A.Z, indicating that exclusion of H2A.Z from methylated DNA is the cause (Figure 6). The bisulfite sequencing data also reveal global decreases in CHG and CHH TE methylation in the *h2a.z* mutant. Changes in TE methylation could be a direct result of H2A.Z loss, or may be caused by a variety of indirect effects. Given the depletion of H2A.Z from methylated transposons and the substantial transcriptional and developmental changes in *h2a.z* plants, we consider indirect explanations to be more probable. For example, the approximately two-fold downregulation of the DNA methyltransferase CMT3, which catalyzes CHG methylation [77], might be partly responsible for the decreased CHG methylation. (Table S5).

### H2A.Z in gene bodies regulates transcription of responsive genes

The significance of H2A.Z enrichment near transcriptional start sites has been a major focus of research [22], [23], [78], [79], [80], but a distinct function for H2A.Z in gene bodies has been recently hypothesized [81]. Consistent with this idea, we previously showed that H2A.Z abundance within gene bodies correlates negatively with transcription in Arabidopsis and the pufferfish *Tetraodon nigroviridis*, whereas H2A.Z near the TSS is most enriched in moderately transcribed genes in both organisms [16], [24]. Human studies also show that gene body H2A.Z correlates with silencing [12] and that H2A.Z is depleted from the bodies of actively transcribed genes [23]. The presence of this relationship in plants and animals implies that it is an ancient property of eukaryotes. Interestingly, recent studies in yeast have shown that mutation of the IN080 complex causes loss of H2A.Z near the TSS and gain of H2A.Z across the coding region [51], suggesting that competing nucleosome remodelers may shape the genic patterns of H2A.Z.

Here, we show that H2A.Z within gene bodies is correlated with gene responsiveness, consistent with recent yeast data demonstrating that H2A.Z is enriched across coding sequences of genes that are differentially transcribed after environmental stress [26]. Loss of H2A.Z leads to misregulation of Arabidopsis genes with high responsiveness scores, which measure differential expression across both tissue types and environmental conditions. Furthermore, this misregulation occurs despite a lack of change in the DNA methylation profiles of these genes in the *h2a.z* mutant. Our results are consistent with evidence from many other species, where loss of H2A.Z leads to misregulation of various inducible genes, including environmental response genes in yeast [21], [26], [78] and developmentally regulated and tissue-specific genes in animals [13], [15], [18], [82], [83]. The phenotypes of *h2a.z* mutants, including altered flowering time, floral homeotic transformations and silique deformation, also strongly imply that developmental regulators are misregulated. We also demonstrate that genes that show little change in transcription in our *h2a.z* mutant plants tend to have H2A.Z depleted from the gene body, whereas those genes with either strong up- or downregulation tend to have much more gene-body H2A.Z. Taken together, these results indicate that H2A.Z within transcribed sequences is necessary for proper regulation of responsive genes but may antagonize constitutive and high-level expression, and that this relationship is both ancient and well-conserved across many eukaryotic lineages.

### Gene body methylation may regulate gene expression by preventing H2A.Z incorporation

The presence of DNA methylation within the bodies of animal and plant genes has been known for some time [84], [85]. Recent genome-wide bisulfite sequencing in various eukaryotic species has revealed that gene body methylation is an ancient and widely conserved feature of eukaryotic chromatin predating the divergence of animals and plants [24], [38], [43], [44], [45], [71], [86], [87]. In animals and plants, gene body methylation exists almost exclusively within the CG context and follows a consistent pattern, with depletion of DNA methylation from the 5′ and 3′ ends of genes. Taken together with the finding that many species of invertebrates have DNA methylation primarily or exclusively within gene bodies [24], [87], [88], these results strongly suggest that genic methylation plays an important and conserved function in at least some eukaryotic lineages [89].

Despite the prevalence of gene body methylation in diverse eukaryotes, its function remains mysterious [90]. A potential clue comes from the correlation between genic methylation and transcription. Gene body methylation is highest in moderately transcribed genes in plants and animals, with the lowest levels of genic methylation at either transcriptional extreme [24], [45], [71]. Additionally, there is an unexplained negative linear correlation between genic methylation and gene responsiveness in Arabidopsis and the honeybee *Apis mellifera* [70], [71], [91]. High levels of body methylation tend to be found in slowly evolving genes with vital housekeeping functions in honeybee, silkworm (*Bombyx mori*), sea squirt (*Ciona intestinalis*), sea anemone (*Nematostella vectensis*), poplar (*Populus tricharpa*), and Arabidopsis [71], [88], [92], [93]. These results indicate that DNA methylation of the transcribed region may be important for proper regulation of constitutively expressed genes.

Here, we show that the genome-wide anticorrelation between DNA methylation and H2A.Z is established by the exclusion of H2A.Z from methylated DNA. Because gene body DNA methylation and H2A.Z show opposing correlations with gene responsiveness, and the anticorrelation between DNA methylation and H2A.Z is ancient, we propose that a basal function of genic DNA methylation is the stabilization of constitutive expression patterns within housekeeping genes by antagonizing H2A.Z deposition (Figure 6). As H2A.Z has been linked to the regulation of inducible genes in many organisms, including species such as *S. cerevisae* and *C. elegans* that lack DNA methylation [8], [13], [15], [18], [21], [26], [69], [78], [82], [94], and DNA methylation can exclude H2A.Z but not vice versa, we believe that the presence or absence of H2A.Z in the gene body is a better candidate for direct gene regulation than DNA methylation. The functional significance of DNA methylation of constitutive genes may be primarily to prevent incorporation of H2A.Z.

## Materials and Methods

### Biological materials

The Arabidopsis T-DNA lines *hta9-1* (SALK_054814), *hta11-1* (SALK_017235), *ibm1–6* (SALK_006042), and *pie1–5* (SALK_096434) were obtained from the SALK collection (*Col-0* ecotype) (http://signal.salk.edu/). The Arabidopsis T-DNA line *hta8-1* (FLAG_593B04) was obtained from the INRA (http://www-ijpb.versailles.inra.fr/) collection (*WS* ecotype). Sequencing of the 5′ promoter region of *HTA8* confirmed the T-DNA insertion site for *hta8-1* at position 16,220,917 on Chr2 (NC_003071.1), 8 bp downstream of the 5′ end of gene model AT2G38810.2. The Arabidopsis EMS mutant *met1–6* is described in [95] (*Col-0* ecotype). The *h2a.z* mutant was generated from crosses of an *hta9-1*; *hta-11* double mutant with the *hta8-1* line. In all experiments, the WT control line for each mutant was generated from the nearest WT sibling of the mutant (i.e. the WT control for the *h2a.z* mutant is an F2 progeny of wild-type genotype at all three H2A.Z loci, derived from an F1 parent that is heterozygous for *hta9-1*, *hta11-1*, and *hta8-1*). In instances where we felt it was necessary to distinguish which WT control we refer to (i.e. Figure S6), we use the non-mutant gene identifier (in all capital letters) preceding the “WT” (i.e., the WT line associated with the *pie1* mutant is “PIE1 WT”). For bisulfite sequencing of seedling tissues, seeds were planted on 1× Murashige and Skoog Media with micronutrients and 1.5% Sucrose (Caisson Laboratories) and grown under 16 h light/8 h dark for 14 days in a growth chamber. For bisulfite sequencing of rosette and cauline leaf tissue, seeds were planted on soil and grown in greenhouse conditions with LD 16 h light/8 h dark. For phenotype analysis of the *h2a.z* mutant, seeds were planted on soil and grown in greenhouse conditions with either 16 h light/8 h dark (LD) or 8 h light/16 h dark (SD). Genotyping of SALK and INRA T-DNA lines was carried out by PCR with primers listed in Table S6. Genotyping of the *met1–6* line was carried out by dCAPS-PCR with primers listed in Table S6 and subsequent digestion with *BglII*.

### Transcript analysis of H2A.Z genes

Expression analyses for the *h2a.z* and *hta9-1* mutant lines and for the WT control were performed on total RNA extracted from 4 week post germination rosette leaves grown on soil in LD conditions using the RNeasy Plant Extraction Kit (Qiagen) with the optional on-column DNAse treatment. RT-PCR reactions were carried out on cDNA generated using 1 ug total RNA and the Superscript III Kit (Invitrogen) using gene specific primers listed in Table S6. qPCR was carried out on similarly generated cDNA using EvaGreen Detection chemistry on an ABI 7500 FAST Real-Time PCR System with primers in exons flanking the single intron in *HTA9*. The gene *UBQ5* (AT3G62250) was used as in internal control. Three biological replicates, each with three technical replicates, were averaged.

### Bisulfite sequencing

Approximately 100–500 ng genomic DNA was isolated from either seedling, rosette or cauline leaf tissues. Seedling tissue was obtained from 14 days post germination seedlings grown on Murashige and Skoog media in LD (16 h light/8 h dark). Mature rosette leaves and mature cauline leaves were obtained from 4 week post germination mature plants grown on soil in LD (16 h light/8 h dark). In general, multiple biological replicates were generated for each mutant and WT line; a complete list of all generated libraries is available in Table S7. WT datasets for each mutant were generated from plants derived from recent relatives of the relevant mutant. Bisulfite conversion and Illumina library construction and sequencing were performed as described in [96]. We used single ends (SE) Illumina sequencing for bisulfite sequencing on the GAII and HiSeq platforms and sequence alignments were performed using Bowtie [97] and the TAIR8 Genome Annotation (http://www.arabidopsis.org/) as in [24]. The average percent methylation plots were generated as described in [96] and [24].

### Locus-specific methylation analysis

For locus-specific bisulfite sequencing (referred to as BS-PCR), data were generated exactly as described previously [16]. Bisulfite-converted genomic DNA from 14 day seedlings was PCR amplified using primers from each of four genotypes, including *h2a.z*, *pie1*, H2A.Z WT, and PIE1 WT. Approximately 10–12 clones were sequenced from each genotype (except for At3g22340, in which 6 clones were sequenced for *pie1*) and percent methylation was determined at the same cytosine sites used for this calculation in our previous publication. Alternatively, percent methylation scores were also calculated by extracting the reads associated with each locus from the relevant whole genome bisulfite sequencing datasets (referred to as BS-Seq). Average percent methylation levels were calculated at the same cytosine sites described above for BS-PCR from available seedling replicates for each genotype (2 for *pie1*, 3 for PIE1 WT, 2 for *h2a.z*, 2 for H2A.Z WT).

### RNA sequencing

Approximately 30 ug total RNA was isolated from 4 week post germination mature rosette leaves using the RNeasy Plant Extraction Kit (Qiagen) with the optional on-column DNAse treatment. mRNA was purified from total RNA by two treatments of poly-A enrichment using the Oligotex kit (Qiagen #72022), followed by a rRNA removal step using the RiboMinus Plant Kit for RNA sequencing (Invitrogen #A1083702). Illumina library construction and RNA sequencing were performed as described in [24]. We used single ends (SE) Illumina sequencing for RNA sequencing on the GAII platform and sequence alignments were performed using Bowtie and the TAIR8 Genome Annotation and cDNA Annotation (http://www.arabidopsis.org/) as in [24].

### H2A.Z ChAP sequencing

H2A.Z-containing nucleosomes were chromatin affinity purified (ChAP) from 4 week post germination Arabidopsis roots of our H2A.Z-BLRP transgenic lines grown in LD conditions as in [16]. Illumina libraries were constructed for IP and input DNA samples and sequenced on the HiSeq 2000 platform, generating 50 bp reads. Sequence alignments were performed using Bowtie and the TAIR8 genome annotation as in [24]. Nucleosomal midpoints were estimated based on an average 150-bp nucleosome length by adding 75 bp to the start position of each read. Differences between IP and input over each single-base window were generated to give an overall genome-wide map of H2A.Z-enrichment.

### Differential expression sequence analysis

For differential expression analysis of the RNA sequencing datasets, a strategy was employed to account for expression differences between WS and Col ecotypes. In brief, we used the recently published list of 144,879 SNPs between the WS and Col ecotypes [98] to obtain reads per kilobase of exon model per million reads (RPKM) scores for each gene in *h2a.z* and WT from either the WS or Col backgrounds.

First, using Bowtie with no tolerance for mismatches, reads from each of the three *h2a.z* and WT RNA sequencing datasets were mapped to small 75 bp scaffolds containing either the WS or Col SNP around each SNP locus that mapped within an exon of a gene greater than 200 bp in length and with at least 10 mapped reads. We removed all SNPs that were less than one read-length (36 bp) from the end of the exon, which left approximately 5,000 SNPs across the genome. The number of reads mapping to the WS and Col scaffolds were compared at each SNP locus and used to determine whether the region was homozygous for WS, Col or heterozygous for the two ecotypes in each dataset. For SNPs at heterozygous loci, a Read Count Contribution from each WS or Col genome was determined by dividing the number of reads mapping to either WS or Col genome by the total reads mapping to the SNP scaffold for each ecotype. As SNPs within a given heterozygous region generally exhibited similar ratios of WS to Col mapped reads, a rolling 20-window (where the windows are the 5,000 SNPs) smoothing function was applied to these read count contribution values.

Next, the six RNA sequencing datasets were mapped to the TAIR cDNA scaffold, and each cDNA model was assigned a score equal to the number of mapped RPKM. For both the *h2a.z* and WT datasets, the normalized read counts of the three replicates were partitioned into reads contributed by WS and by Col using the smoothed read count contribution value obtained from the nearest SNP. In this way, approximate WS and Col read count scores were determined for each gene in both *h2a.z* and WT.

To test for statistical significance of the difference between the *h2a.z* and WT, we repeated the above partitioning process using read counts normalized to the size of the smallest library, rather than per million of reads. This alternate normalization less drastically underestimates the number of reads per locus, which is important as the statistical significance is dependent on the number of reads. We calculated the probability that a gene's expression deviates from expectation using a Fisher's two-tailed exact test of *h2a.z* vs. WT scores for each ecotype. Genes were determined to be differentially expressed if for either ecotype they exhibited a two-fold change in expression between *h2a.z* and WT and had a P-value<0.001, or if for both ecotypes they exhibited a two-fold change in expression and had p-values<0.005. Gene Ontology analysis was performed on the up- and downregulated gene lists using the GO FAT Ontology on the DAVID web server (http://david.abcc.ncifcrf.gov) [99], [100] and categories with P-values<1×10^−5^ were considered enriched.

### Data deposition

Sequences are deposited in Gene Expression Omnibus (GEO) with accession number GSE39045.

## Supporting Information

Figure S1 Residual expression of *HTA9* in the *h2a.z* mutant. Sequencing of residual *HTA9* transcripts in the *h2a.z* mutant. Genomic DNA sequence of the 15 bp (5 amino acids) on either side of the exon/intron boundary are shown in black. The WT and *h2a.z* mRNA transcripts from the corresponding region are shown in green and red, respectively.
(TIF)

Figure S2 Phenotypes of the *h2a.z* mutant grown in long and short day conditions. Phenotypes of *h2a.z* mutant and WT plants at 28 days post germination grown in LD conditions (A) and 40 days post germination grown in SD conditions (B).
(TIF)

Figure S3 Rare developmental phenotypes of the *h2a.z* mutant. (A) and (B) Inappropriate emergence of petals and stamens directly from stem structures. (C) Abnormal floral morphology with ovules presented externally. (D) Transformation of outer sepal into floral inflorescence. (E) Phyllotaxy defects. (F) Abnormal leaf morphology.
(TIF)

Figure S4 Loss of H2A.Z does not substantially affect genic DNA methylation. Profiles of CG, CHG, and CHH DNA methylation in *h2a.z* and WT, for two independent replicates of seedlings (A–B), rosettes (C–D), and cauline leaves (E–F). Genes were aligned as in Figure 3 and average methylation levels for each 100-bp interval are plotted from 2 kb away from the gene (negative numbers) to 5 kb into the gene (positive numbers). WT methylation is represented by the green traces, while *h2a.z* methylation is represented by red traces. The Y-axis was partitioned at 0.017 and the lower portion expanded to aid in the visibility of CHG and CHH traces.
(TIF)

Figure S5 Loss of PIE1 does not substantially affect genic DNA methylation. Profiles of CG, CHG, and CHH DNA methylation in (A) for one replicate of *pie1*, *h2a.z;pie1*, and WT, and an additional replicate each of *pie1* and WT in (B). Genes were aligned as in Figure 3 and average methylation levels for each 100-bp interval are plotted from 2 kb away from the gene (negative numbers) to 5 kb into the gene (positive numbers). WT methylation is represented by the green traces; *pie1* methylation is represented by brown traces; *h2a.z;pie1* methylation is represented by yellow traces. The Y-axis was partitioned at 0.017 and the lower portion expanded to aid in the visibility of CHG and CHH traces.
(TIF)

Figure S6 Locus-specific analyses of methylation in *h2a.z* and *pie1.* Bar graphs of average percent methylation for five selected loci: At1g69850 (a nitrate transporter), At3g22340 (a COPIA-like retrotransposon), At4g03480 (an ankyrin-repeat-containing protein), At4g38190 (a cellulose synthase) and At5g37450 (a protein kinase). Percent methylation scores were determined by either locus-specific bisulfite sequencing (BS-PCR) or through *in silico* extraction of relevant reads from the whole-genome bisulfite sequencing datasets (BS-Seq) (see Methods). (A) For each locus, the data from left to right are: previously published *pie1* BS-PCR data, current *pie1* BS-PCR data, current *pie1* BS-Seq data, previous PIE1 WT BS-PCR data, current PIE1 WT BS-PCR data, and current PIE1 WT BS-Seq data. (B) For each locus, the data from left to right are: previously published *pie1* BS-PCR data, current *h2a.z* BS-PCR data, current *h2a.z* BS-Seq data, previous PIE1 WT BS-PCR data, current H2A.Z WT BS-PCR data, and current H2A.ZWT BS-Seq data.
(TIF)

Figure S7 Methylation differences between *h2a.z*-related mutants and WT. Frequency plots of the occurrence of 50 bp windows with differential methylation between mutant and WT in genes, plotted by their p-value significance. Separate plots are shown for the windows in genes with low H2A.Z enrichment in gene bodies, genes with high H2A.Z enrichment in gene bodies, and the combined total of these two categories. P-values were determined with a modified Fisher's Exact test (using total “c” and “t” counts for pooled mutant or WT datasets). Windows which showed less than a 10% difference in methylation in either direction between mutant and WT, or which overlapped with transposon annotations, were excluded. Frequency counts for windows with greater methylation in the mutant than WT are shown in red, while counts for windows with greater methylation in WT than in mutant are shown in green.
(TIF)

Figure S8 The *h2a.z* mutant exhibits changes in transposon methylation. Profiles of CG, CHG, and CHH DNA methylation in *h2a.z* and WT, for two independent replicates of seedlings (A–B), rosettes (C–D), and cauline leaves (E–F). Transposons were aligned as in Figure 3 and average methylation levels for each 100-bp interval are plotted from 2 kb away from the TE (negative numbers) to 5 kb into the TE (positive numbers). WT methylation is represented by the green traces, while *h2a.z* methylation is represented by red traces.
(TIF)

Figure S9 *h2a.z* induces global changes in transposon methylation. Kernel density plots, which have the effect of tracing the frequency distribution, for the differences between *h2a.z* and WT CG (A), CHG (B), and CHH (C) methylation in TEs for seedlings (blue traces), rosettes (green traces) and cauline leaves (red traces). The distributions of methylation differences for each 50 bp window located within TEs and having average levels of methylation greater than zero in both *h2a.z* and WT are shown.
(TIF)

Figure S10 *h2a.z;pie1* causes greater loss of transposon methylation than either *h2a.z* or *pie1*. (A) Profiles of CG, CHG, and CHH DNA methylation in TEs in seedlings for one replicate each of *pie1*, *h2a.z;pie1* and WT. TEs were aligned as in Figure 3 and average methylation levels for each 100-bp interval are plotted. WT methylation is represented by green traces, *pie1* by brown traces, and *h2a.z;pie1* by yellow traces. An additional replicate each of *pie1* and WT are shown in (B). (C) Box plots of differences in CG, CHG and CHH methylation between WT and either *h2a.z*, *pie1* or *h2a.z;pie1* seedlings for all 50 bp windows within TEs. Each box encloses the middle 50% of the distribution, with the horizontal black line marking the median. The lines extending vertically from each box mark the minimum and maximum values that fall within 1.5 times the height of the box.
(TIF)

Figure S11 Genic CG DNA methylation profiles of H2A.Z-deficient and DNA methylation-perturbed double mutants. (A) Profiles of CG DNA methylation in *h2a.z*, *h2a.z;met1*, *met1* and WT. Genes were aligned as in Figure 3 and average methylation levels for each 100-bp interval are plotted from 2 kb away from the gene to 5 kb into the gene. WT methylation is represented by the green trace, *h2a.z* methylation by the red trace, *met1* methylation by the purple trace, and *h2a.z;met1* methylation by the pink trace. The dashed line at zero represents the point of alignment. (B) Profiles of CG DNA methylation in genes, as in (A), for *h2a.z* (red trace), *h2a.z;ibm1* (light blue trace), *ibm1* (dark blue trace) and WT (green trace). (C) Profiles of CG DNA methylation in genes, as in (A), for *pie1* (brown trace), *pie1;ibm1* (beige trace), *ibm1* (dark blue trace) and WT (green trace).
(TIF)

Figure S12 Genic CHG DNA methylation profiles of H2A.Z-deficient and DNA methylation-perturbed double mutants. (A) Profiles of CHG DNA methylation in *h2a.z*, *h2a.z;met1*, *met1* and WT. Genes were aligned as in Figure 3 and average methylation levels for each 100-bp interval are plotted from 2 kb away from the gene to 5 kb into the gene. WT methylation is represented by the green trace, *h2a.z* methylation by the red trace, *met1* methylation by the purple trace, and *h2a.z;met1* methylation by the pink trace. The dashed line at zero represents the point of alignment. (B) Profiles of CHG DNA methylation in genes, as in (A), for *h2a.z* (red trace), *h2a.z;ibm1* (light blue trace), *ibm1* (dark blue trace) and WT (green trace). (C) Profiles of CHG DNA methylation in genes, as in (A), for *pie1* (brown trace), *pie1;ibm1* (beige trace), *ibm1* (dark blue trace) and WT (green trace). Note the difference in scale for figures (B) and (C), due to differences in *ibm1* CHG hypermethylation between the 1st and 2nd generation plants (discussed in the text).
(TIF)

Figure S13 Genic CHH DNA methylation profiles of H2A.Z-deficient and DNA methylation-perturbed double mutants. (A) Profiles of CHH DNA methylation in *h2a.z*, *h2a.z;met1*, *met1* and WT. Genes were aligned as in Figure 3 and average methylation levels for each 100-bp interval are plotted from 2 kb away from the gene to 5 kb into the gene. WT methylation is represented by the green trace, *h2a.z* methylation by the red trace, *met1* methylation by the purple trace, and *h2a.z;met1* methylation by the pink trace. The dashed line at zero represents the point of alignment. (B) Profiles of CHH DNA methylation in genes, as in (A), for *h2a.z* (red trace), *h2a.z;ibm1* (light blue trace), *ibm1* (dark blue trace) and WT (green trace). (C) Profiles of CHH DNA methylation in genes, as in (A), for *pie1* (brown trace), *pie1;ibm1* (beige trace), *ibm1* (dark blue trace) and WT (green trace).
(TIF)

Figure S14 Methylation differences between double mutants and control lines. Frequency plots of the occurrence of 50 bp windows with differential methylation between double mutant and parental control lines in genes, plotted by their p-value significance as in Figure S7. Separate plots are shown for the windows in genes with low H2A.Z in gene bodies, genes with high H2A.Z in gene bodies, and the combined total. Frequency counts for windows with greater methylation in the double mutant than the control are shown in red, while counts for windows with greater methylation in the control than in the double mutant are shown in green.
(TIF)

Figure S15 Transposon CG DNA methylation profiles of H2A.Z-deficient and DNA methylation-perturbed double mutants. (A) Profiles of CG DNA methylation in *h2a.z;met1*, *h2a.z*, *met1* and WT. Transposons were aligned as in Figure 3 and average methylation levels for each 100-bp interval are plotted from 2 kb away from the TE to 5 kb into the TE. WT methylation is represented by the green trace, *h2a.z* methylation by the red trace, *met1* methylation by the purple trace, and *h2a.z;met1* methylation by the pink trace. The dashed line at zero represents the point of alignment. (B) Profiles of CG DNA methylation in TEs, as in (A), for *h2a.z* (red trace), *h2a.z;ibm1* (light blue trace), *ibm1* (dark blue trace) and WT (green trace). (C) Profiles of CG DNA methylation in TEs, as in (A), for *pie1* (brown trace), *pie1;ibm1* (beige trace), *ibm1* (dark blue trace) and WT (green trace).
(TIF)

Figure S16 CHG hypomethylation of transposons is greater in *pie1;ibm1* than in parental lines. (A) Profiles of CHG DNA methylation in *pie1* (brown trace), *pie1;ibm1* (beige trace), and *ibm1* (dark blue trace). Transposons were aligned as in Figure 3 and average methylation levels for each 100-bp interval are plotted from 2 kb away from the TE to 5 kb into the TE. The dashed line at zero represents the point of alignment.
(TIF)

Figure S17 H2A.Z-deficient and DNA methylation-perturbed double mutants show greater loss of TE CHH methylation. (A) Profiles of CHH DNA methylation in *h2a.z;met1*, *met1* and WT. Transposons were aligned as in Figure 3 and average methylation levels for each 100-bp interval are plotted from 2 kb away from the TE to 5 kb into the TE. WT methylation is represented by the green trace, *met1* methylation by the purple trace, and *h2a.z;met1* methylation by the pink trace. The dashed line at zero represents the point of alignment. (B) Profiles of CHH DNA methylation in TEs, as in (A) for *h2a.z;ibm1* (light blue trace), *ibm1* (dark blue trace) and WT (green trace). (C) Profiles of CHH DNA methylation in TEs, as in (A), for *pie1* (brown trace), *pie1;ibm1* (beige trace), and WT (green trace).
(TIF)

Figure S18 Comparisons of H2A.Z ChAP Sequencing and ChIP-chip data. Heat maps of H2A.Z enrichment across genes in various datasets. (A) Our current H2A.Z ChAP sequencing data, presented exactly as in Figure 4C (for reference). The data were clustered into 9 approximately equal sized groups based on three tiers (low, mid, high) of H2A.Z enrichment at the TSS and across the body (see Figure 4C). (B) Our previous H2A.Z ChIP-chip data from [16], presented using the same clustering as in (A). (C) Our previous ChIP-chip data for H2A.Z enrichment in *met1* [16], presented using the same clustering as in (A), shown here as (*met1* – WT) to aid in visualization of changes that occur in the *met1* mutant.
(TIF)

Figure S19 DNA methylation profiles of *h2a.z* and WT grouped by H2A.Z enrichment. Profiles of CG DNA methylation in the *h2a.z* and WT datasets, for (A) genes with low H2A.Z at the TSS (n = 3,916), (B) genes with high H2A.Z at the TSS (n = 4,086), (C) genes with low H2A.Z across the gene body (n = 3,920), and (D) genes with high H2A.Z across the gene body (n = 4,081). Genes were aligned as in Figure 3. WT methylation is represented by the green traces, while *h2a.z* methylation is represented by red traces. The dashed lines at zero represents the point of alignment.
(TIF)

Figure S20 H2A.Z enrichment and methylation in hypervariable and housekeeping genes. (A–C) Average profiles of H2A.Z enrichment (IP - input) across gene bodies. Genes were aligned at the 5′ end (left half of panel) and the 3′ end (right half of panel) and average H2A.Z enrichment levels for each 50-bp interval are plotted from 2 kb away from the gene (negative numbers) to 2 kb into the gene (positive numbers). To avoid averaging H2A.Z enrichment at the 5′ and 3′ ends into the H2A.Z body distribution, we do not use data within 1 kb of the opposite end of the gene. Plots show H2A.Z enrichment in the 1000 most responsive genes as defined in [70], and 1000 randomly selected genes with responsiveness score of 0 (least responsive genes) grouped by length: 2–3 kb in (A), 3–4 kb in (B) and 4–5 kb in (C). The dashed lines at zero represent the points of alignment. (D) Average profiles of H2A.Z enrichment in hypervariable genes and housekeeping genes, as defined in [70], between 1.5 and 3 kb in length. Genes were aligned as in (A–C), except that the alignment was extended 1.5 kb into the gene. (E) DNA methylation in hypervariable and housekeeping genes. Genes were aligned at the 5′ end (left half of panel) and the 3′ end (right half of panel) and average methylation levels for each 100-bp interval are plotted from 1 kb away from the gene (negative numbers) to 3 kb into the gene (positive numbers).
(TIF)

Figure S21 *h2a.z*, *pie1*, and *hta9;hta11* transcriptional misregulation. Venn diagrams for genes upregulated (A) or downregulated (B) in *h2a.z*, *pie1*, and *hta9;hta11*. The *h2a.z* data are taken from the RNA sequencing presented here, while both the *pie1* and *hta9;hta11* data are from previously published lists of misregulated genes in these mutants [37]. Genes are defined here as upregulated if the Log_2_ (mutant/WT) score is >0.5 (upregulated) or <−0.5 (downregulated). P-values for the associated overlaps between each pair of mutants, calculated using a modified Fisher's Exact test, are indicated outside the Venn diagram where each pair of circles meet.
(TIF)

Table S1 H2A.Z Body-Depleted Genes. List of genes (n = 3,920) that have low levels of H2A.Z enriched within gene bodies, as defined in the text.
(XLS)

Table S2 H2A.Z Body-Enriched Genes. List of genes (n = 4,081) that have high levels of H2A.Z enriched within gene bodies, as defined in the text.
(XLS)

Table S3 Overrepresented GO terms in upregulated genes in the *h2a.z* mutant. List of all overrepresented GO Terms in the 1800 upregulated genes in the *h2a.z* mutant, as determined by DAVID using the GO Term BP FAT Ontology Categories. From left to right are the GO Term ID, GO Term, Fold Enrichment, P-Value, Gene Count, and the percentage that Gene Count is out of the total number of upregulated genes. Shown in bold are all of the GO Terms with “response” in their name.
(XLS)

Table S4 Overrepresented GO terms in downregulated genes in the *h2a.z* mutant. List of all overrepresented GO Terms in the 544 downregulated genes in the *h2a.z* mutant, as determined by DAVID using the GO Term BP FAT Ontology Categories. From left to right are the GO Term ID, GO Term, Fold Enrichment, P-Value, Gene Count, and the percentage that Gene Count is out of the total number of downregulated genes. Shown in bold are all of the GO Terms with “response” in their name.
(XLS)

Table S5 Transcription of methylation factors in *h2a.z*. List of transcriptional changes (Log_2_ (*h2a.z*/WT) RMKM) for genes involved in DNA methylation of genes and TEs.
(XLS)

Table S6 Primers. List of all primers used for genotyping, RT-PCR, or qPCR in this study. Columns (in order) represent gene name, TAIR ID, primer name, primer type, and primer sequence (5′ to 3′).
(XLS)

Table S7 Illumina sequencing libraries. List of all sequencing libraries generated in this study. Columns (in order) represent the genotype of the library material, replicate number, library name, library type, tissue type, and age of tissue.
(XLS)

## References

[1] Talbert PB, Henikoff S (2010) Histone variants–ancient wrap artists of the epigenome. Nat Rev Mol Cell Biol 11: 264–275. 20197778 10.1038/nrm2861

[2] Henikoff S, Ahmad K (2005) Assembly of variant histones into chromatin. Annu Rev Cell Dev Biol 21: 133–153. 16212490 10.1146/annurev.cellbio.21.012704.133518

[3] Sarma K, Reinberg D (2005) Histone variants meet their match. Nat Rev Mol Cell Biol 6: 139–149. 15688000 10.1038/nrm1567

[4] Barzily-Rokni M, Friedman N, Ron-Bigger S, Isaac S, Michlin D, et al (2011) Synergism between DNA methylation and macroH2A1 occupancy in epigenetic silencing of the tumor suppressor gene p16(CDKN2A). Nucleic Acids Res 39: 1326–1335. 21030442 10.1093/nar/gkq994 PMC3045621

[5] Zlatanova J, Thakar A (2008) H2A.Z: view from the top. Structure 16: 166–179. 18275809 10.1016/j.str.2007.12.008

[6] Brickner DG, Cajigas I, Fondufe-Mittendorf Y, Ahmed S, Lee PC, et al (2007) H2A.Z-mediated localization of genes at the nuclear periphery confers epigenetic memory of previous transcriptional state. PLoS Biol 5: e81 doi:10.1371/journal.pbio.0050081. 17373856 10.1371/journal.pbio.0050081 PMC1828143

[7] Light WH, Brickner DG, Brand VR, Brickner JH (2010) Interaction of a DNA zip code with the nuclear pore complex promotes H2A.Z incorporation and INO1 transcriptional memory. Mol Cell 40: 112–125. 20932479 10.1016/j.molcel.2010.09.007 PMC2953765

[8] Kumar SV, Wigge PA (2010) H2A.Z-containing nucleosomes mediate the thermosensory response in Arabidopsis. Cell 140: 136–147. 20079334 10.1016/j.cell.2009.11.006

[9] Dhillon N, Oki M, Szyjka SJ, Aparicio OM, Kamakaka RT (2006) H2A.Z functions to regulate progression through the cell cycle. Mol Cell Biol 26: 489–501. 16382141 10.1128/MCB.26.2.489-501.2006 PMC1346916

[10] Li B, Pattenden SG, Lee D, Gutierrez J, Chen J, et al (2005) Preferential occupancy of histone variant H2AZ at inactive promoters influences local histone modifications and chromatin remodeling. Proc Natl Acad Sci U S A 102: 18385–18390. 16344463 10.1073/pnas.0507975102 PMC1317944

[11] Guillemette B, Bataille AR, Gevry N, Adam M, Blanchette M, et al (2005) Variant histone H2A.Z is globally localized to the promoters of inactive yeast genes and regulates nucleosome positioning. PLoS Biol 3: e384 doi:10.1371/journal.pbio.0030384. 16248679 10.1371/journal.pbio.0030384 PMC1275524

[12] Barski A, Cuddapah S, Cui K, Roh TY, Schones DE, et al (2007) High-resolution profiling of histone methylations in the human genome. Cell 129: 823–837. 17512414 10.1016/j.cell.2007.05.009

[13] Creyghton MP, Markoulaki S, Levine SS, Hanna J, Lodato MA, et al (2008) H2AZ is enriched at polycomb complex target genes in ES cells and is necessary for lineage commitment. Cell 135: 649–661. 18992931 10.1016/j.cell.2008.09.056 PMC2853257

[14] Albert I, Mavrich TN, Tomsho LP, Qi J, Zanton SJ, et al (2007) Translational and rotational settings of H2A.Z nucleosomes across the Saccharomyces cerevisiae genome. Nature 446: 572–576. 17392789 10.1038/nature05632

[15] Whittle CM, McClinic KN, Ercan S, Zhang X, Green RD, et al (2008) The genomic distribution and function of histone variant HTZ-1 during C. elegans embryogenesis. PLoS Genet 4: e1000187 doi:10.1371/journal.pgen.1000187. 18787694 10.1371/journal.pgen.1000187 PMC2522285

[16] Zilberman D, Coleman-Derr D, Ballinger T, Henikoff S (2008) Histone H2A.Z and DNA methylation are mutually antagonistic chromatin marks. Nature 456: 125–129. 18815594 10.1038/nature07324 PMC2877514

[17] Siegel TN, Hekstra DR, Kemp LE, Figueiredo LM, Lowell JE, et al (2009) Four histone variants mark the boundaries of polycistronic transcription units in Trypanosoma brucei. Genes Dev 23: 1063–1076. 19369410 10.1101/gad.1790409 PMC2682952

[18] Petter M, Lee CC, Byrne TJ, Boysen KE, Volz J, et al (2011) Expression of P. falciparum var genes involves exchange of the histone variant H2A.Z at the promoter. PLoS Pathog 7: e1001292 doi:10.1371/journal.ppat.1001292. 21379342 10.1371/journal.ppat.1001292 PMC3040674

[19] Raisner RM, Hartley PD, Meneghini MD, Bao MZ, Liu CL, et al (2005) Histone variant H2A.Z marks the 5′ ends of both active and inactive genes in euchromatin. Cell 123: 233–248. 16239142 10.1016/j.cell.2005.10.002 PMC2039754

[20] Zhang H, Roberts DN, Cairns BR (2005) Genome-wide dynamics of Htz1, a histone H2A variant that poises repressed/basal promoters for activation through histone loss. Cell 123: 219–231. 16239141 10.1016/j.cell.2005.08.036 PMC2788555

[21] Millar CB, Xu F, Zhang K, Grunstein M (2006) Acetylation of H2AZ Lys 14 is associated with genome-wide gene activity in yeast. Genes Dev 20: 711–722. 16543223 10.1101/gad.1395506 PMC1413291

[22] Mavrich TN, Jiang C, Ioshikhes IP, Li X, Venters BJ, et al (2008) Nucleosome organization in the Drosophila genome. Nature 453: 358–362. 18408708 10.1038/nature06929 PMC2735122

[23] Hardy S, Jacques PE, Gevry N, Forest A, Fortin ME, et al (2009) The euchromatic and heterochromatic landscapes are shaped by antagonizing effects of transcription on H2A.Z deposition. PLoS Genet 5: e1000687 doi:10.1371/journal.pgen.1000687. 19834540 10.1371/journal.pgen.1000687 PMC2754525

[24] Zemach A, McDaniel IE, Silva P, Zilberman D (2010) Genome-wide evolutionary analysis of eukaryotic DNA methylation. Science 328: 916–919. 20395474 10.1126/science.1186366

[25] Wan Y, Saleem RA, Ratushny AV, Roda O, Smith JJ, et al (2009) Role of the histone variant H2A.Z/Htz1p in TBP recruitment, chromatin dynamics, and regulated expression of oleate-responsive genes. Mol Cell Biol 29: 2346–2358. 19273605 10.1128/MCB.01233-08 PMC2668375

[26] Sadeghi L, Bonilla C, Stralfors A, Ekwall K, Svensson JP (2011) Podbat: a novel genomic tool reveals Swr1-independent H2A.Z incorporation at gene coding sequences through epigenetic meta-analysis. PLoS Comput Biol 7: e1002163 doi:10.1371/journal.pcbi.1002163. 21901086 10.1371/journal.pcbi.1002163 PMC3161910

[27] Jackson JD, Gorovsky MA (2000) Histone H2A.Z has a conserved function that is distinct from that of the major H2A sequence variants. Nucleic Acids Res 28: 3811–3816. 11000274 10.1093/nar/28.19.3811 PMC110762

[28] Santisteban MS, Kalashnikova T, Smith MM (2000) Histone H2A.Z regulats transcription and is partially redundant with nucleosome remodeling complexes. Cell 103: 411–422. 11081628 10.1016/s0092-8674(00)00133-1

[29] Kobor MS, Venkatasubrahmanyam S, Meneghini MD, Gin JW, Jennings JL, et al (2004) A protein complex containing the conserved Swi2/Snf2-related ATPase Swr1p deposits histone variant H2A.Z into euchromatin. PLoS Biol 2: e131 doi:10.1371/journal.pbio.0020131. 15045029 10.1371/journal.pbio.0020131 PMC374244

[30] Krogan NJ, Baetz K, Keogh MC, Datta N, Sawa C, et al (2004) Regulation of chromosome stability by the histone H2A variant Htz1, the Swr1 chromatin remodeling complex, and the histone acetyltransferase NuA4. Proc Natl Acad Sci U S A 101: 13513–13518. 15353583 10.1073/pnas.0405753101 PMC518788

[31] Mizuguchi G, Shen X, Landry J, Wu WH, Sen S, et al (2004) ATP-driven exchange of histone H2AZ variant catalyzed by SWR1 chromatin remodeling complex. Science 303: 343–348. 14645854 10.1126/science.1090701

[32] Noh YS, Amasino RM (2003) PIE1, an ISWI family gene, is required for FLC activation and floral repression in Arabidopsis. Plant Cell 15: 1671–1682. 12837955 10.1105/tpc.012161 PMC165409

[33] Choi K, Park C, Lee J, Oh M, Noh B, et al (2007) Arabidopsis homologs of components of the SWR1 complex regulate flowering and plant development. Development 134: 1931–1941. 17470967 10.1242/dev.001891

[34] Deal RB, Topp CN, McKinney EC, Meagher RB (2007) Repression of flowering in Arabidopsis requires activation of FLOWERING LOCUS C expression by the histone variant H2A.Z. Plant Cell 19: 74–83. 17220196 10.1105/tpc.106.048447 PMC1820970

[35] Ruhl DD, Jin J, Cai Y, Swanson S, Florens L, et al (2006) Purification of a human SRCAP complex that remodels chromatin by incorporating the histone variant H2A.Z into nucleosomes. Biochemistry 45: 5671–5677. 16634648 10.1021/bi060043d

[36] Wong MM, Cox LK, Chrivia JC (2007) The chromatin remodeling protein, SRCAP, is critical for deposition of the histone variant H2A.Z at promoters. J Biol Chem 282: 26132–26139. 17617668 10.1074/jbc.M703418200

[37] March-Diaz R, Garcia-Dominguez M, Lozano-Juste J, Leon J, Florencio FJ, et al (2008) Histone H2A.Z and homologues of components of the SWR1 complex are required to control immunity in Arabidopsis. Plant J 53: 475–487. 17988222 10.1111/j.1365-313X.2007.03361.x

[38] Feng S, Cokus SJ, Zhang X, Chen PY, Bostick M, et al (2010) Conservation and divergence of methylation patterning in plants and animals. Proc Natl Acad Sci U S A 107: 8689–8694. 20395551 10.1073/pnas.1002720107 PMC2889301

[39] Glastad KM, Hunt BG, Yi SV, Goodisman MA (2011) DNA methylation in insects: on the brink of the epigenomic era. Insect Mol Biol 20: 553–565. 21699596 10.1111/j.1365-2583.2011.01092.x

[40] Law JA, Jacobsen SE (2010) Establishing, maintaining and modifying DNA methylation patterns in plants and animals. Nat Rev Genet 11: 204–220. 20142834 10.1038/nrg2719 PMC3034103

[41] Zhang X, Jacobsen SE (2006) Genetic Analyses of DNA Methyltransferases in Arabidopsis thaliana. Cold Spring Harb Symp Quant Biol 71: 439–447. 17381326 10.1101/sqb.2006.71.047

[42] Vaughn MW, Tanurd Ic M, Lippman Z, Jiang H, Carrasquillo R, et al (2007) Epigenetic Natural Variation in Arabidopsis thaliana. PLoS Biol 5: e174 doi:10.1371/journal.pbio.0050174. 17579518 10.1371/journal.pbio.0050174 PMC1892575

[43] Cokus SJ, Feng S, Zhang X, Chen Z, Merriman B, et al (2008) Shotgun bisulphite sequencing of the Arabidopsis genome reveals DNA methylation patterning. Nature 452: 215–219. 18278030 10.1038/nature06745 PMC2377394

[44] Lister R, O'Malley RC, Tonti-Filippini J, Gregory BD, Berry CC, et al (2008) Highly Integrated Single-Base Resolution Maps of the Epigenome in Arabidopsis. Cell 133: 523–536. 18423832 10.1016/j.cell.2008.03.029 PMC2723732

[45] Zilberman D, Gehring M, Tran RK, Ballinger T, Henikoff S (2007) Genome-wide analysis of Arabidopsis thaliana DNA methylation uncovers an interdependence between methylation and transcription. Nat Genet 39: 61–69. 17128275 10.1038/ng1929

[46] He XJ, Chen T, Zhu JK (2011) Regulation and function of DNA methylation in plants and animals. Cell Res 21: 442–465. 21321601 10.1038/cr.2011.23 PMC3152208

[47] Conerly ML, Teves SS, Diolaiti D, Ulrich M, Eisenman RN, et al (2010) Changes in H2A.Z occupancy and DNA methylation during B-cell lymphomagenesis. Genome Res 20: 1383–1390. 20709945 10.1101/gr.106542.110 PMC2945187

[48] Edwards JR, O'Donnell AH, Rollins RA, Peckham HE, Lee C, et al (2010) Chromatin and sequence features that define the fine and gross structure of genomic methylation patterns. Genome Res 20: 972–980. 20488932 10.1101/gr.101535.109 PMC2892098

[49] Auger A, Galarneau L, Altaf M, Nourani A, Doyon Y, et al (2008) Eaf1 is the platform for NuA4 molecular assembly that evolutionarily links chromatin acetylation to ATP-dependent exchange of histone H2A variants. Mol Cell Biol 28: 2257–2270. 18212047 10.1128/MCB.01755-07 PMC2268442

[50] Bowman TA, Wong MM, Cox LK, Baldassare JJ, Chrivia JC (2011) Loss of H2A.Z Is Not Sufficient to Determine Transcriptional Activity of Snf2-Related CBP Activator Protein or p400 Complexes. Int J Cell Biol 2011: 715642. 21785598 10.1155/2011/715642 PMC3140016

[51] Papamichos-Chronakis M, Watanabe S, Rando OJ, Peterson CL (2011) Global regulation of H2A.Z localization by the INO80 chromatin-remodeling enzyme is essential for genome integrity. Cell 144: 200–213. 21241891 10.1016/j.cell.2010.12.021 PMC3035940

[52] Wu WH, Alami S, Luk E, Wu CH, Sen S, et al (2005) Swc2 is a widely conserved H2AZ-binding module essential for ATP-dependent histone exchange. Nat Struct Mol Biol 12: 1064–1071. 16299513 10.1038/nsmb1023

[53] Redon C, Pilch D, Rogakou E, Sedelnikova O, Newrock K, et al (2002) Histone H2A variants H2AX and H2AZ. Curr Opin Genet Dev 12: 162–169. 11893489 10.1016/s0959-437x(02)00282-4

[54] Yi H, Sardesai N, Fujinuma T, Chan CW, Veena, et al (2006) Constitutive expression exposes functional redundancy between the Arabidopsis histone H2A gene HTA1 and other H2A gene family members. Plant Cell 18: 1575–1589. 16751347 10.1105/tpc.105.039719 PMC1488917

[55] Matsuda R, Hori T, Kitamura H, Takeuchi K, Fukagawa T, et al (2010) Identification and characterization of the two isoforms of the vertebrate H2A.Z histone variant. Nucleic Acids Res 38: 4263–4273. 20299344 10.1093/nar/gkq171 PMC2910051

[56] Faast R, Thonglairoam V, Schulz TC, Beall J, Wells JR, et al (2001) Histone variant H2A.Z is required for early mammalian development. Curr Biol 11: 1183–1187. 11516949 10.1016/s0960-9822(01)00329-3

[57] Rangasamy D, Berven L, Ridgway P, Tremethick DJ (2003) Pericentric heterochromatin becomes enriched with H2A.Z during early mammalian development. EMBO J 22: 1599–1607. 12660166 10.1093/emboj/cdg160 PMC152904

[58] Ridgway P, Brown KD, Rangasamy D, Svensson U, Tremethick DJ (2004) Unique residues on the H2A.Z containing nucleosome surface are important for Xenopus laevis development. J Biol Chem 279: 43815–43820. 15299007 10.1074/jbc.M408409200

[59] van Daal VH, van der Leij A (1992) Computer-based reading and spelling practice for children with learning disabilities. J Learn Disabil 25: 186–195. 1602226 10.1177/002221949202500306

[60] Deal RB, Kandasamy MK, McKinney EC, Meagher RB (2005) The nuclear actin-related protein ARP6 is a pleiotropic developmental regulator required for the maintenance of FLOWERING LOCUS C expression and repression of flowering in Arabidopsis. Plant Cell 17: 2633–2646. 16141450 10.1105/tpc.105.035196 PMC1242262

[61] Morillo-Huesca M, Clemente-Ruiz M, Andujar E, Prado F (2010) The SWR1 histone replacement complex causes genetic instability and genome-wide transcription misregulation in the absence of H2A.Z. PLoS ONE 5: e12143 doi:10.1371/journal.pone.0012143. 20711347 10.1371/journal.pone.0012143 PMC2920830

[62] Hang M, Smith MM (2011) Genetic analysis implicates the Set3/Hos2 histone deacetylase in the deposition and remodeling of nucleosomes containing H2A.Z. Genetics 187: 1053–1066. 21288874 10.1534/genetics.110.125419 PMC3070515

[63] Saze H, Shiraishi A, Miura A, Kakutani T (2008) Control of genic DNA methylation by a jmjC domain-containing protein in Arabidopsis thaliana. Science 319: 462–465. 18218897 10.1126/science.1150987

[64] Miura A, Nakamura M, Inagaki S, Kobayashi A, Saze H, et al (2009) An Arabidopsis jmjC domain protein protects transcribed genes from DNA methylation at CHG sites. EMBO J 28: 1078–1086. 19262562 10.1038/emboj.2009.59 PMC2653724

[65] Finnegan EJ, Dennis ES (1993) Isolation and identification by sequence homology of a putative cytosine methyltransferase from Arabidopsis thaliana. Nucleic Acids Res 21: 2383–2388. 8389441 10.1093/nar/21.10.2383 PMC309536

[66] Kankel MW, Ramsey DE, Stokes TL, Flowers SK, Haag JR, et al (2003) Arabidopsis MET1 cytosine methyltransferase mutants. Genetics 163: 1109–1122. 12663548 10.1093/genetics/163.3.1109 PMC1462485

[67] Saze H, Mittelsten Scheid O, Paszkowski J (2003) Maintenance of CpG methylation is essential for epigenetic inheritance during plant gametogenesis. Nat Genet 34: 65–69. 12669067 10.1038/ng1138

[68] Inagaki S, Kakutani T (2010) Control of genic DNA methylation in Arabidopsis. J Plant Res 123: 299–302. 20364290 10.1007/s10265-010-0338-1

[69] Smith AP, Jain A, Deal RB, Nagarajan VK, Poling MD, et al (2009) Histone H2A.Z regulates the expression of several classes of phosphate starvation response genes but not as a transcriptional activator. Plant Physiol 152: 217–225. 19897606 10.1104/pp.109.145532 PMC2799358

[70] Aceituno FF, Moseyko N, Rhee SY, Gutierrez RA (2008) The rules of gene expression in plants: organ identity and gene body methylation are key factors for regulation of gene expression in Arabidopsis thaliana. BMC Genomics 9: 438. 18811951 10.1186/1471-2164-9-438 PMC2566314

[71] Zhang X, Yazaki J, Sundaresan A, Cokus S, Chan SW, et al (2006) Genome-wide High-Resolution Mapping and Functional Analysis of DNA Methylation in Arabidopsis. Cell 126: 1189–1201. 16949657 10.1016/j.cell.2006.08.003

[72] Foret S, Kucharski R, Pittelkow Y, Lockett GA, Maleszka R (2009) Epigenetic regulation of the honey bee transcriptome: unravelling the nature of methylated genes. BMC Genomics 10: 472. 19828049 10.1186/1471-2164-10-472 PMC2768749

[73] Carr AM, Dorrington SM, Hindley J, Phear GA, Aves SJ, et al (1994) Analysis of a histone H2A variant from fission yeast: evidence for a role in chromosome stability. Mol Gen Genet 245: 628–635. 7808414 10.1007/BF00282226

[74] Liu X, Li B, Ma Gorovsky (1996) Essential and nonessential histone H2A variants in Tetrahymena thermophila. Mol Cell Biol 16: 4305–4311. 8754831 10.1128/mcb.16.8.4305 PMC231429

[75] Swaminathan J, Baxter EM, Corces VG (2005) The role of histone H2Av variant replacement and histone H4 acetylation in the establishment of Drosophila heterochromatin. Genes Dev 19: 65–76. 15630020 10.1101/gad.1259105 PMC540226

[76] Fritsch O, Benvenuto G, Bowler C, Molinier J, Hohn B (2004) The INO80 protein controls homologous recombination in Arabidopsis thaliana. Mol Cell 16: 479–485. 15525519 10.1016/j.molcel.2004.09.034

[77] Lindroth AM, Cao X, Jackson JP, Zilberman D, McCallum CM, et al (2001) Requirement of CHROMOMETHYLASE3 for Maintenance of CpXpG Methylation. Science 10: 10. 10.1126/science.1059745 11349138

[78] Adam M, Robert F, Larochelle M, Gaudreau L (2001) H2A.Z is required for global chromatin integrity and for recruitment of RNA polymerase II under specific conditions. Mol Cell Biol 21: 6270–6279. 11509669 10.1128/MCB.21.18.6270-6279.2001 PMC87352

[79] Gevry N, Chan HM, Laflamme L, Livingston DM, Gaudreau L (2007) p21 transcription is regulated by differential localization of histone H2A.Z. Genes Dev 21: 1869–1881. 17671089 10.1101/gad.1545707 PMC1935026

[80] Jin C, Zang C, Wei G, Cui K, Peng W, et al (2009) H3.3/H2A.Z double variant-containing nucleosomes mark ‘nucleosome-free regions’ of active promoters and other regulatory regions. Nat Genet 41: 941–945. 19633671 10.1038/ng.409 PMC3125718

[81] Fujimoto S, Seebart C, Guastafierro T, Prenni J, Caiafa P, et al (2012) Proteome analysis of protein partners to nucleosomes containing canonical H2A or the variant histones H2A.Z or H2A.X. Biol Chem 10.1515/BC-2011-216 22628298

[82] Amat R, Gudas L (2011) RAR-gamma is Required for Correct Deposition and Removal of Suz12 and H2A.Z in Embryonic Stem Cells. Journal of Cellular Physiology 226: 293–298. 20857416 10.1002/jcp.22420 PMC3369573

[83] Updike DL, Mango SE (2006) Temporal regulation of foregut development by HTZ-1/H2A.Z and PHA-4/FoxA. PLoS Genet 2: e161 doi:10.1371/journal.pgen.0020161. 17009877 10.1371/journal.pgen.0020161 PMC1584275

[84] Tran RK, Henikoff JG, Zilberman D, Ditt RF, Jacobsen SE, et al (2005) DNA methylation profiling identifies CG methylation clusters in Arabidopsis genes. Curr Biol 15: 154–159. 15668172 10.1016/j.cub.2005.01.008

[85] Jones PA, Laird PW (1999) Cancer epigenetics comes of age. Nat Genet 21: 163–167. 9988266 10.1038/5947

[86] Lister R, Pelizzola M, Dowen RH, Hawkins RD, Hon G, et al (2009) Human DNA methylomes at base resolution show widespread epigenomic differences. Nature 462: 315–322. 19829295 10.1038/nature08514 PMC2857523

[87] Xiang H, Zhu J, Chen Q, Dai F, Li X, et al (2010) Single base-resolution methylome of the silkworm reveals a sparse epigenomic map. Nat Biotechnol 28: 516–520. 20436463 10.1038/nbt.1626

[88] Sarda S, Zeng J, Hunt BG, Yi SV (2012) The Evolution of Invertebrate Gene Body Methylation. Mol Biol Evol 10.1093/molbev/mss062 22328716

[89] Suzuki MM, Bird A (2008) DNA methylation landscapes: provocative insights from epigenomics. Nat Rev Genet 9: 465–476. 18463664 10.1038/nrg2341

[90] Zemach A, Zilberman D (2010) Evolution of eukaryotic DNA methylation and the pursuit of safer sex. Curr Biol 20: R780–785. 20833323 10.1016/j.cub.2010.07.007

[91] Zeng J, Yi SV (2010) DNA methylation and genome evolution in honeybee: gene length, expression, functional enrichment covary with the evolutionary signature of DNA methylation. Genome Biol Evol 2: 770–780. 20924039 10.1093/gbe/evq060 PMC2975444

[92] Vining KJ, Pomraning KR, Wilhelm LJ, Priest HD, Pellegrini M, et al (2012) Dynamic DNA cytosine methylation in the Populus trichocarpa genome: tissue-level variation and relationship to gene expression. BMC Genomics 13: 27. 22251412 10.1186/1471-2164-13-27 PMC3298464

[93] Takuno S, Gaut BS (2012) Body-methylated genes in Arabidopsis thaliana are functionally important and evolve slowly. Mol Biol Evol 29: 219–227. 21813466 10.1093/molbev/msr188

[94] Zanton SJ, Pugh BF (2006) Full and partial genome-wide assembly and disassembly of the yeast transcription machinery in response to heat shock. Genes Dev 20: 2250–2265. 16912275 10.1101/gad.1437506 PMC1553208

[95] Xiao W, Gehring M, Choi Y, Margossian L, Pu H, et al (2003) Imprinting of the MEA Polycomb gene is controlled by antagonism between MET1 methyltransferase and DME glycosylase. Dev Cell 5: 891–901. 14667411 10.1016/s1534-5807(03)00361-7

[96] Hsieh TF, Ibarra CA, Silva P, Zemach A, Eshed-Williams L, et al (2009) Genome-wide demethylation of Arabidopsis endosperm. Science 10.1126/science.1172417 PMC4044190 19520962

[97] Langmead B, Trapnell C, Pop M, Salzberg SL (2009) Ultrafast and memory-efficient alignment of short DNA sequences to the human genome. Genome Biol 10: R25. 19261174 10.1186/gb-2009-10-3-r25 PMC2690996

[98] Ashelford K, Eriksson ME, Allen CM, D'Amore R, Johansson M, et al (2011) Full genome re-sequencing reveals a novel circadian clock mutation in Arabidopsis. Genome Biol 12: R28. 21429190 10.1186/gb-2011-12-3-r28 PMC3129678

[99] Huang da W, Sherman BT, Lempicki RA (2009) Systematic and integrative analysis of large gene lists using DAVID bioinformatics resources. Nat Protoc 4: 44–57. 19131956 10.1038/nprot.2008.211

[100] Huang da W, Sherman BT, Lempicki RA (2009) Bioinformatics enrichment tools: paths toward the comprehensive functional analysis of large gene lists. Nucleic Acids Res 37: 1–13. 19033363 10.1093/nar/gkn923 PMC2615629

