# Supplementary figures and images for: Deposition of Histone Variant H2A.Z within Gene Bodies Regulates Responsive Genes
Source: PLoS Genet. 2012 Oct 11;8(10):e1002988. doi: 10.1371/journal.pgen.1002988 (PMC3469445; doi:10.1371/journal.pgen.1002988)

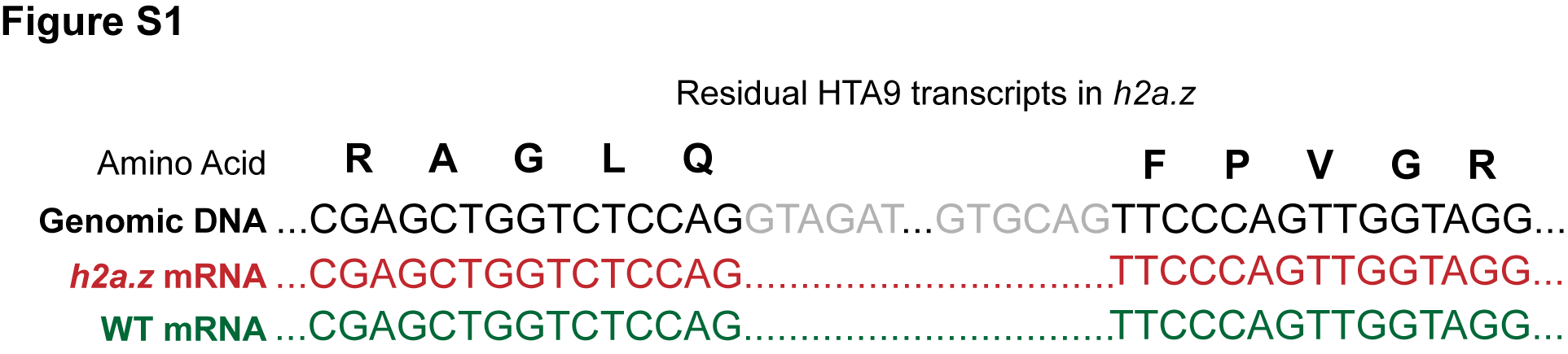

Supplement: Figure S1 — Residual expression of HTA9 in the h2a.z mutant. Sequencing of residual HTA9 transcripts in the h2a.z mutant. Genomic DNA sequence of the 15 bp (5 amino acids) on either side of the exon/intron boundary are shown in black. The WT and h2a.z mRNA transcripts from the corresponding region are shown in green and red, respectively. (TIF) [file pgen.1002988.s001.tif]

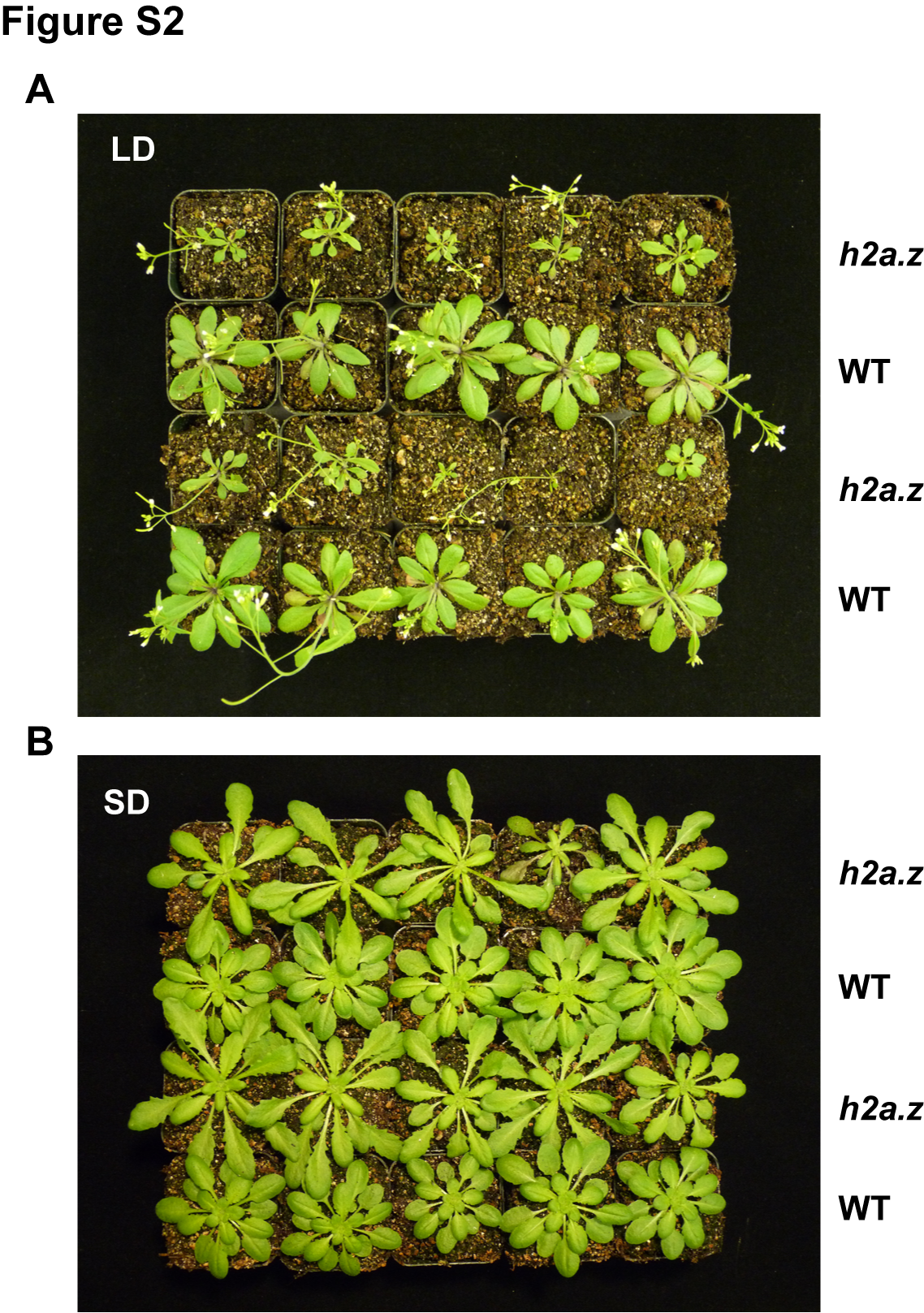

Supplement: Figure S2 — Phenotypes of the h2a.z mutant grown in long and short day conditions. Phenotypes of h2a.z mutant and WT plants at 28 days post germination grown in LD conditions (A) and 40 days post germination grown in SD conditions (B). (TIF) [file pgen.1002988.s002.tif]

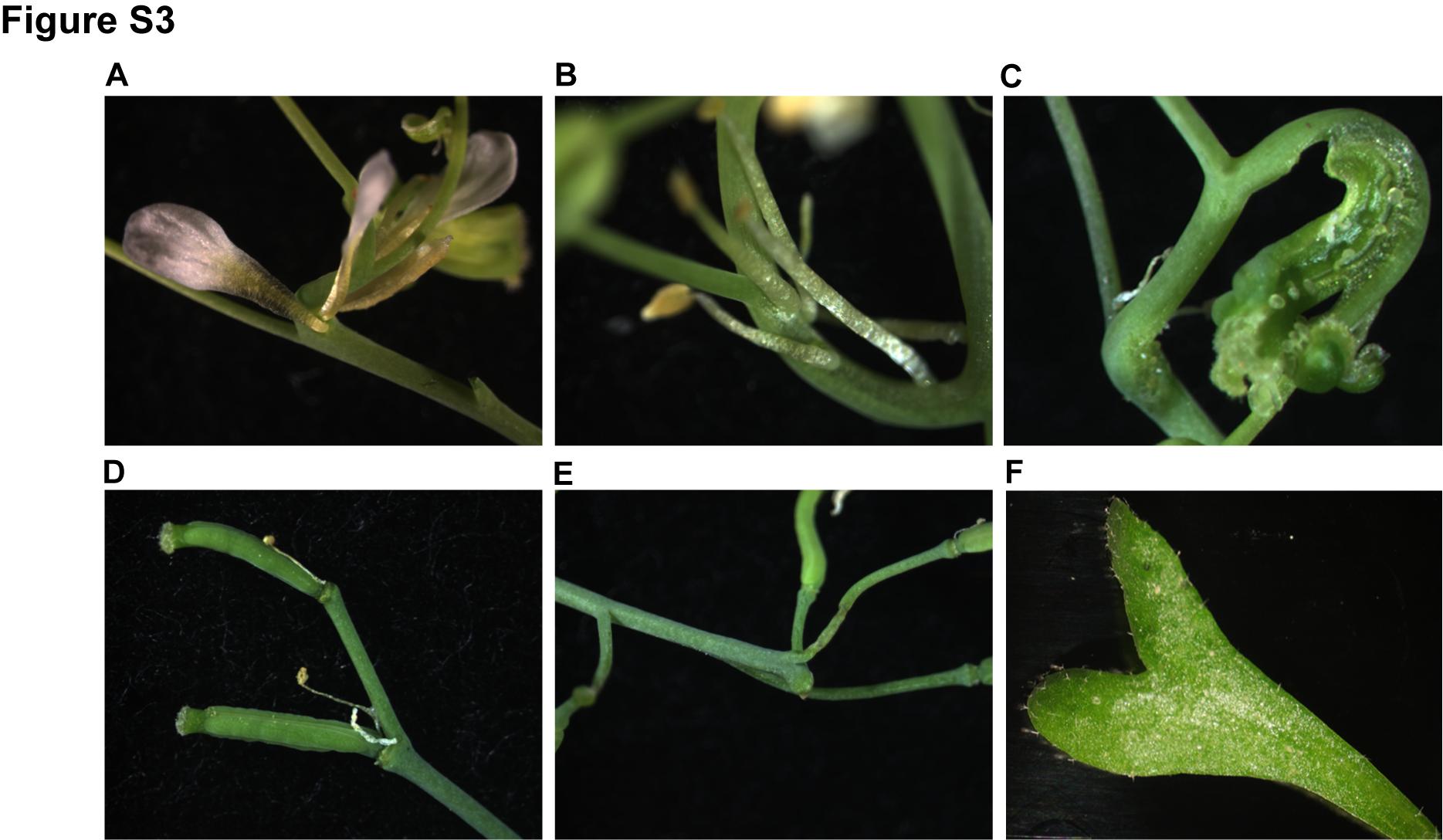

Supplement: Figure S3 — Rare developmental phenotypes of the h2a.z mutant. (A) and (B) Inappropriate emergence of petals and stamens directly from stem structures. (C) Abnormal floral morphology with ovules presented externally. (D) Transformation of outer sepal into floral inflorescence. (E) Phyllotaxy defects. (F) Abnormal leaf morphology. (TIF) [file pgen.1002988.s003.tif]

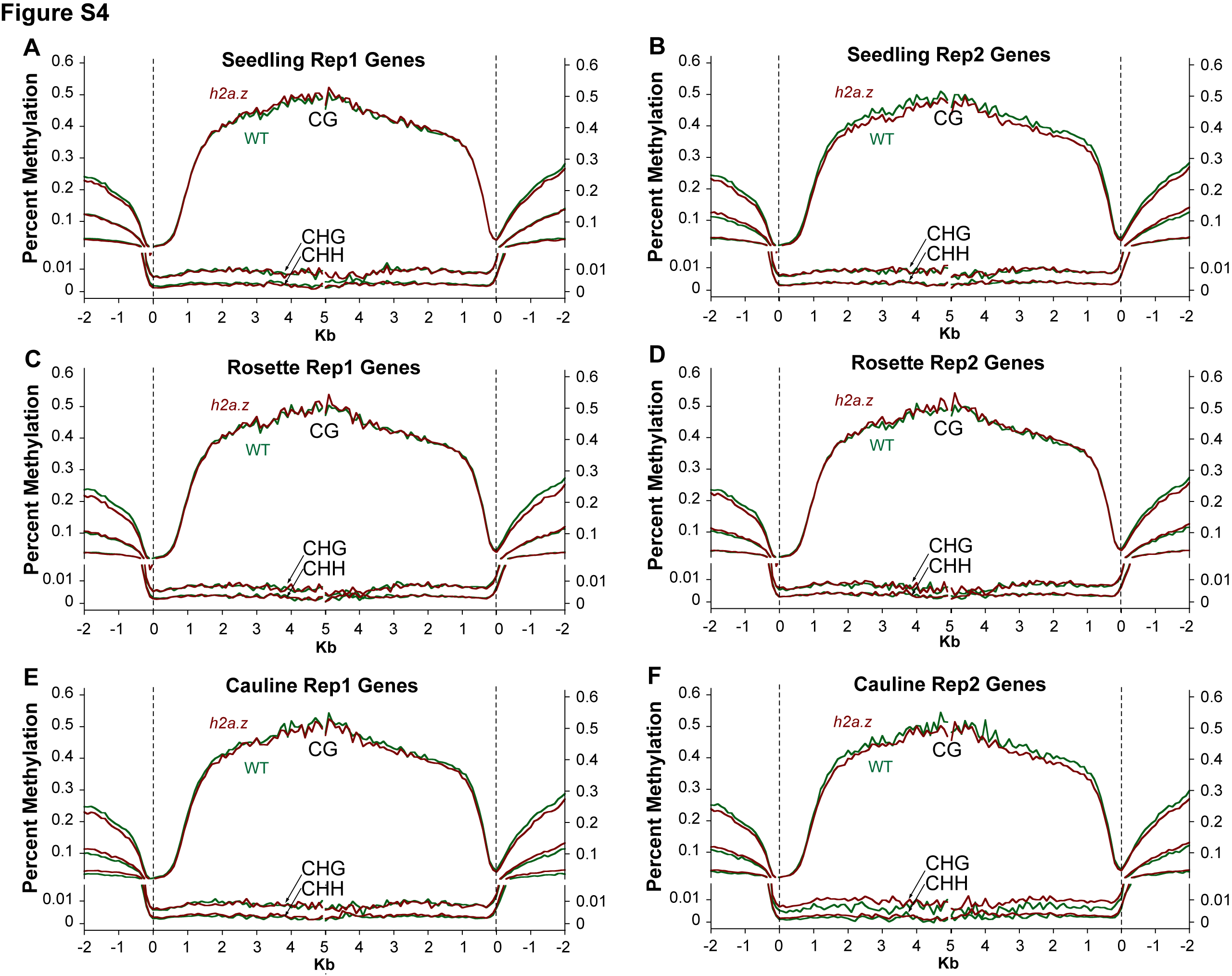

Supplement: Figure S4 — Loss of H2A.Z does not substantially affect genic DNA methylation. Profiles of CG, CHG, and CHH DNA methylation in h2a.z and WT, for two independent replicates of seedlings (A–B), rosettes (C–D), and cauline leaves (E–F). Genes were aligned as in Figure 3 and average methylation levels for each 100-bp interval are plotted from 2 kb away from the gene (negative numbers) to 5 kb into the gene (positive numbers). WT methylation is represented by the green traces, while h2a.z methylation is represented by red traces. The Y-axis was partitioned at 0.017 and the lower portion expanded to aid in the visibility of CHG and CHH traces. (TIF) [file pgen.1002988.s004.tif]

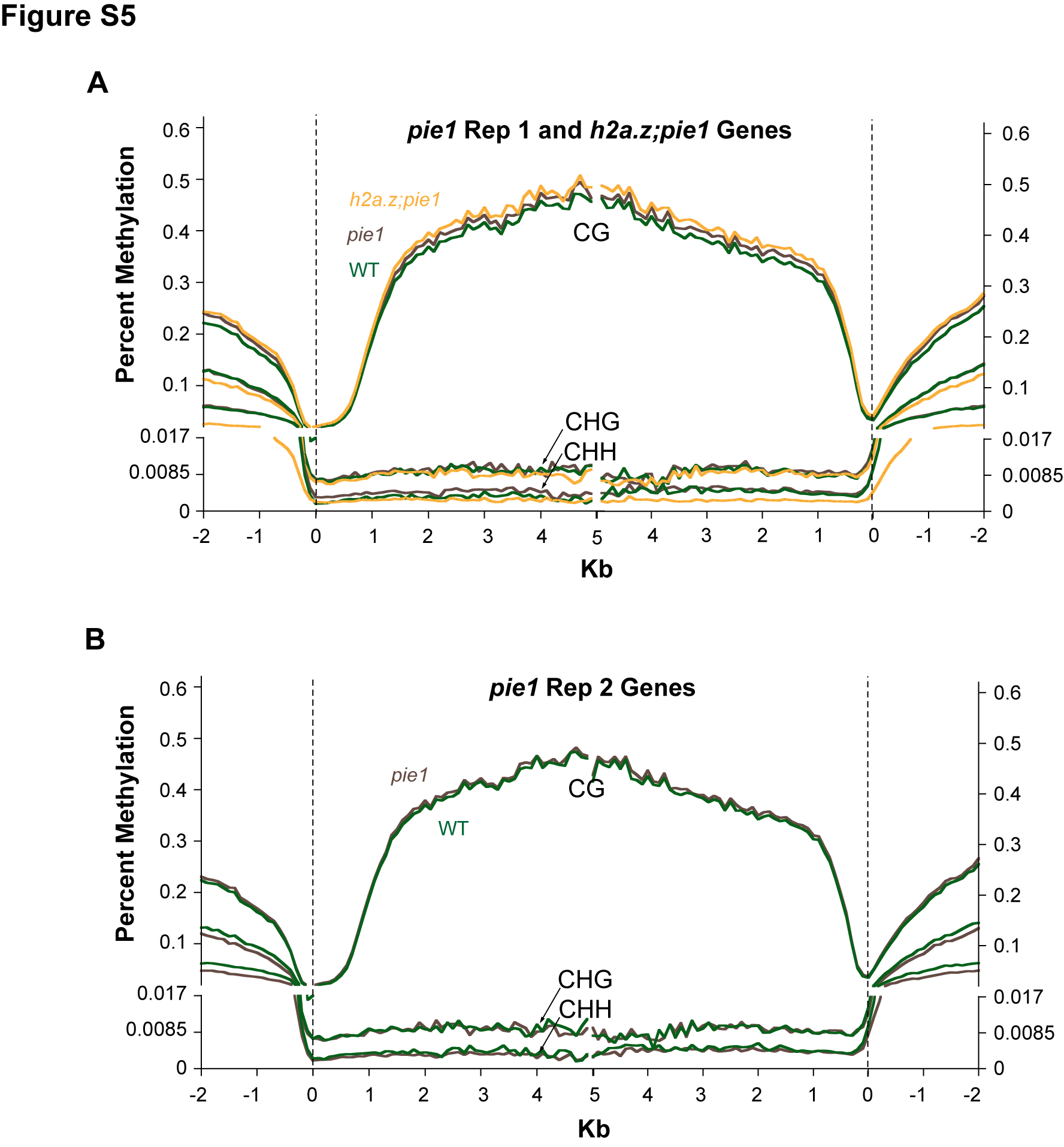

Supplement: Figure S5 — Loss of PIE1 does not substantially affect genic DNA methylation. Profiles of CG, CHG, and CHH DNA methylation in (A) for one replicate of pie1, h2a.z;pie1, and WT, and an additional replicate each of pie1 and WT in (B). Genes were aligned as in Figure 3 and average methylation levels for each 100-bp interval are plotted from 2 kb away from the gene (negative numbers) to 5 kb into the gene (positive numbers). WT methylation is represented by the green traces; pie1 methylation is represented by brown traces; h2a.z;pie1 methylation is represented by yellow traces. The Y-axis was partitioned at 0.017 and the lower portion expanded to aid in the visibility of CHG and CHH traces. (TIF) [file pgen.1002988.s005.tif]

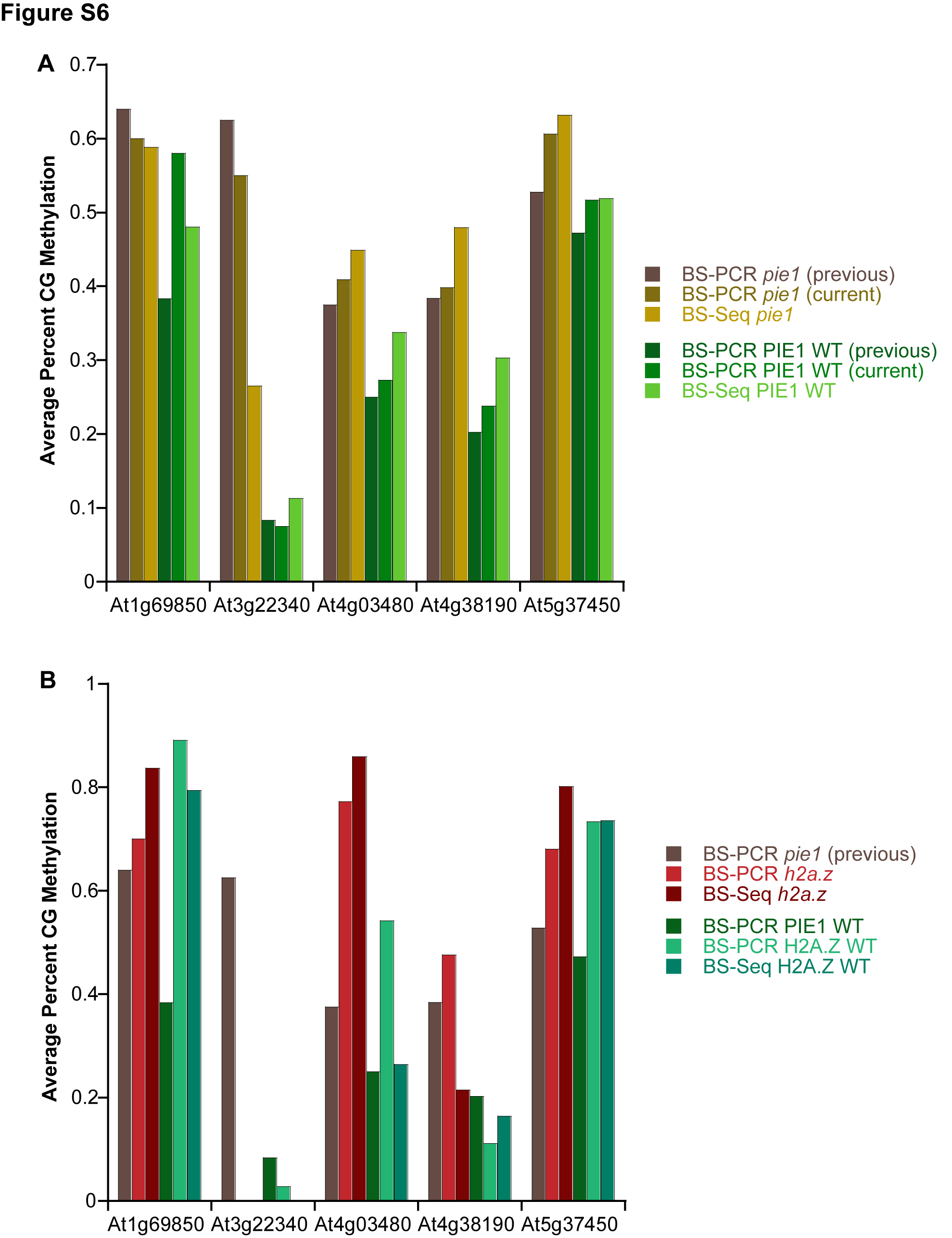

Supplement: Figure S6 — Locus-specific analyses of methylation in h2a.z and pie1. Bar graphs of average percent methylation for five selected loci: At1g69850 (a nitrate transporter), At3g22340 (a COPIA-like retrotransposon), At4g03480 (an ankyrin-repeat-containing protein), At4g38190 (a cellulose synthase) and At5g37450 (a protein kinase). Percent methylation scores were determined by either locus-specific bisulfite sequencing (BS-PCR) or through in silico extraction of relevant reads from the whole-genome bisulfite sequencing datasets (BS-Seq) (see Methods). (A) For each locus, the data from left to right are: previously published pie1 BS-PCR data, current pie1 BS-PCR data, current pie1 BS-Seq data, previous PIE1 WT BS-PCR data, current PIE1 WT BS-PCR data, and current PIE1 WT BS-Seq data. (B) For each locus, the data from left to right are: previously published pie1 BS-PCR data, current h2a.z BS-PCR data, current h2a.z BS-Seq data, previous PIE1 WT BS-PCR data, current H2A.Z WT BS-PCR data, and current H2A.ZWT BS-Seq data. (TIF) [file pgen.1002988.s006.tif]

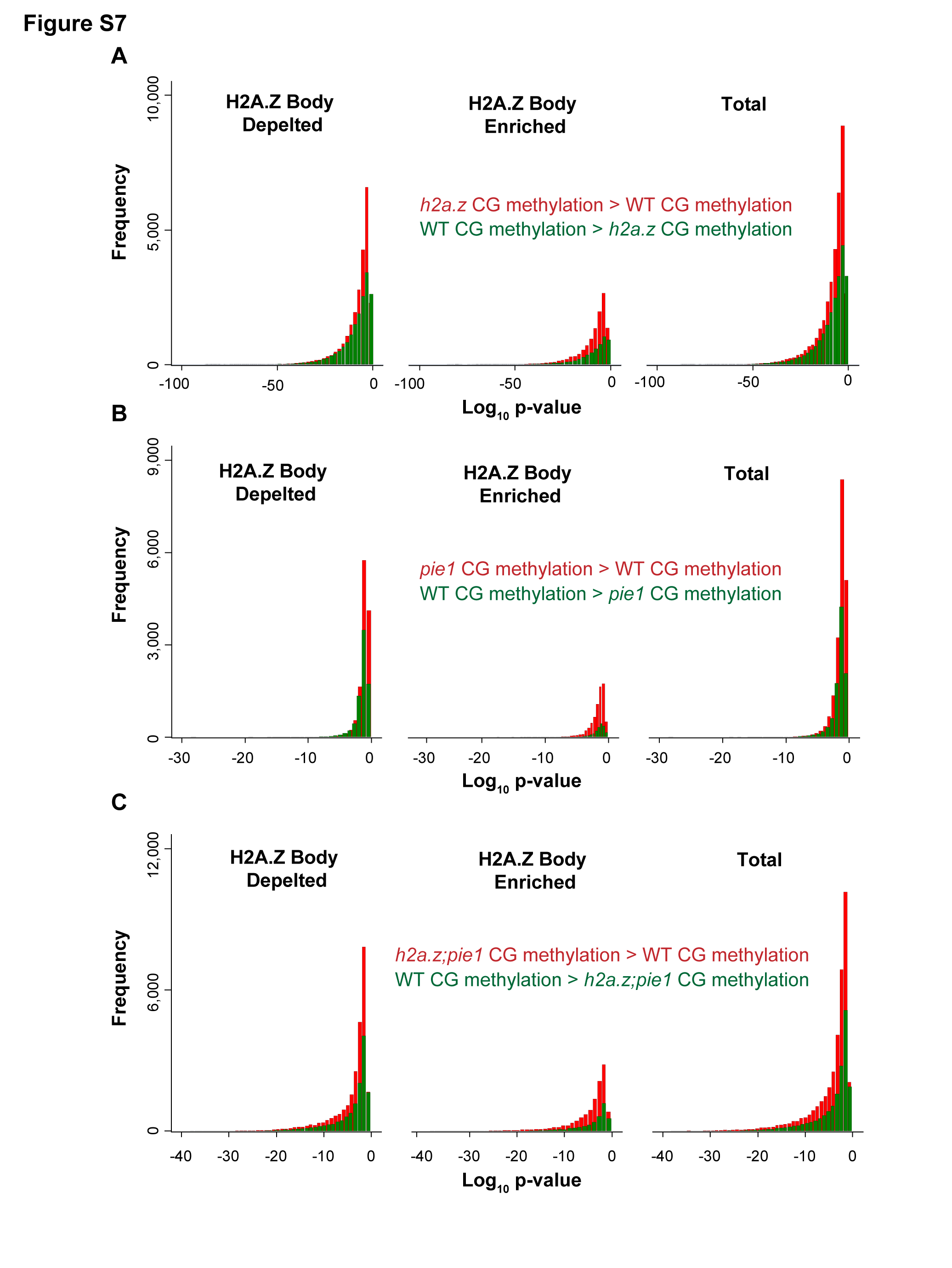

Supplement: Figure S7 — Methylation differences between h2a.z-related mutants and WT. Frequency plots of the occurrence of 50 bp windows with differential methylation between mutant and WT in genes, plotted by their p-value significance. Separate plots are shown for the windows in genes with low H2A.Z enrichment in gene bodies, genes with high H2A.Z enrichment in gene bodies, and the combined total of these two categories. P-values were determined with a modified Fisher's Exact test (using total “c” and “t” counts for pooled mutant or WT datasets). Windows which showed less than a 10% difference in methylation in either direction between mutant and WT, or which overlapped with transposon annotations, were excluded. Frequency counts for windows with greater methylation in the mutant than WT are shown in red, while counts for windows with greater methylation in WT than in mutant are shown in green. (TIF) [file pgen.1002988.s007.tif]

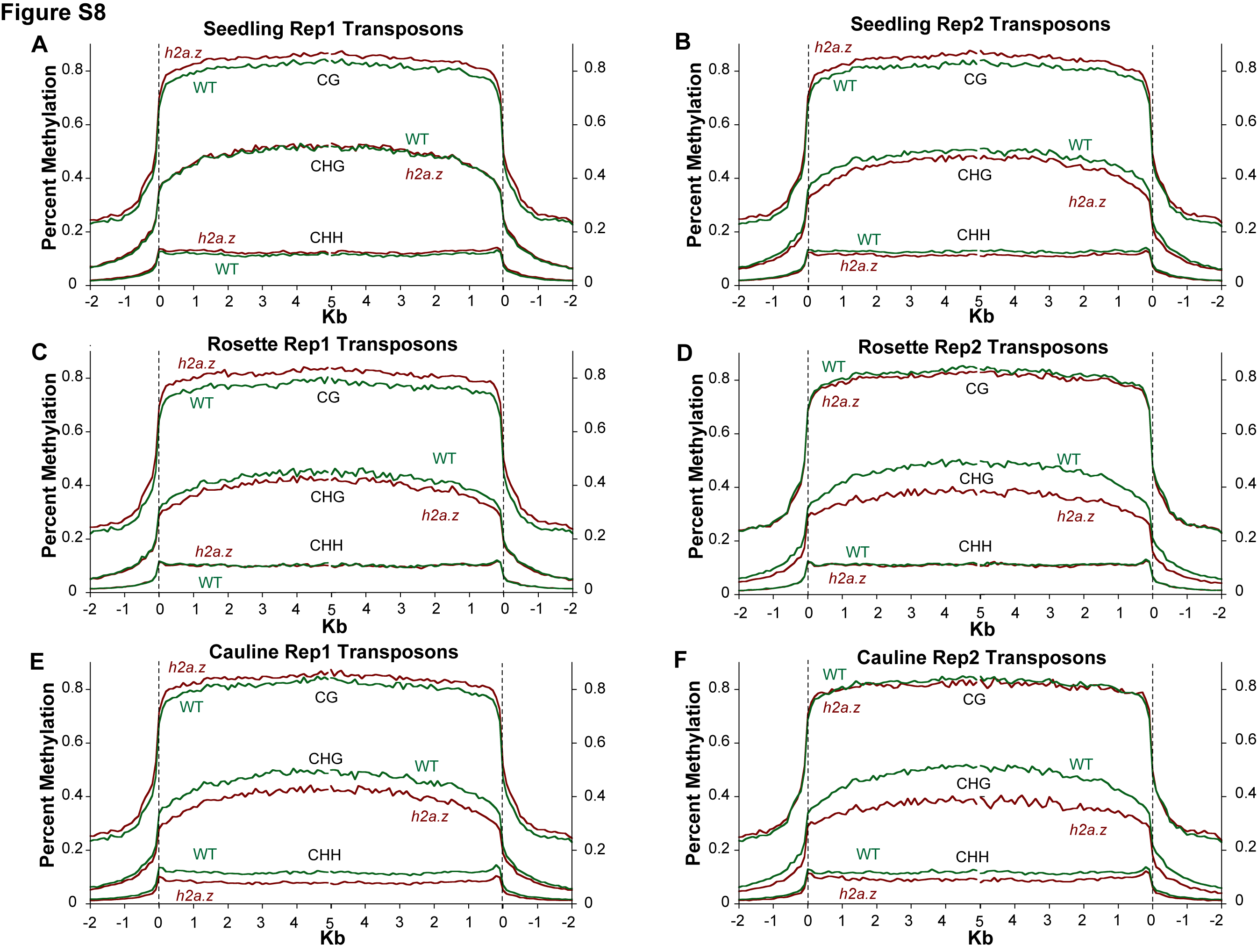

Supplement: Figure S8 — The h2a.z mutant exhibits changes in transposon methylation. Profiles of CG, CHG, and CHH DNA methylation in h2a.z and WT, for two independent replicates of seedlings (A–B), rosettes (C–D), and cauline leaves (E–F). Transposons were aligned as in Figure 3 and average methylation levels for each 100-bp interval are plotted from 2 kb away from the TE (negative numbers) to 5 kb into the TE (positive numbers). WT methylation is represented by the green traces, while h2a.z methylation is represented by red traces. (TIF) [file pgen.1002988.s008.tif]

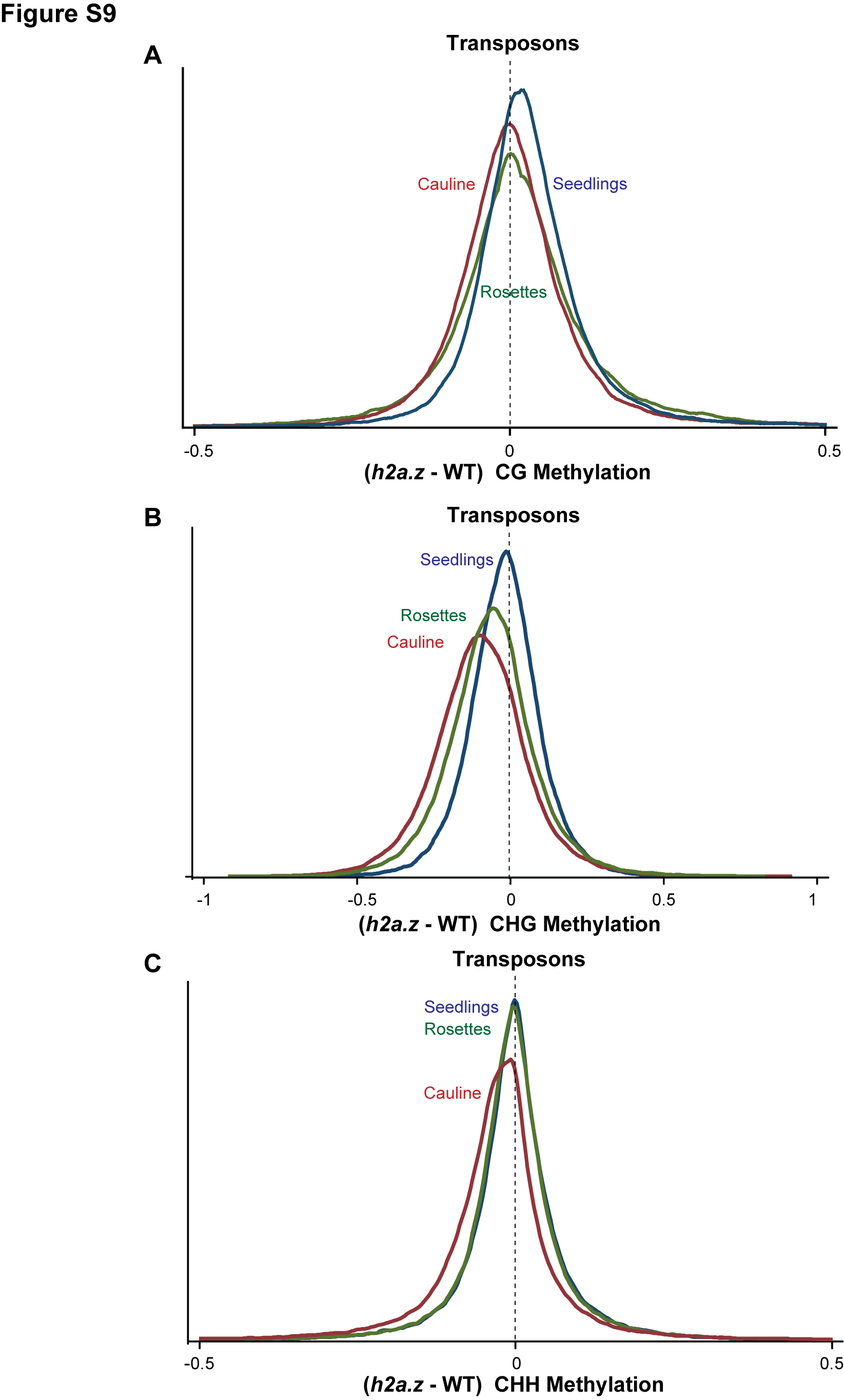

Supplement: Figure S9 — h2a.z induces global changes in transposon methylation. Kernel density plots, which have the effect of tracing the frequency distribution, for the differences between h2a.z and WT CG (A), CHG (B), and CHH (C) methylation in TEs for seedlings (blue traces), rosettes (green traces) and cauline leaves (red traces). The distributions of methylation differences for each 50 bp window located within TEs and having average levels of methylation greater than zero in both h2a.z and WT are shown. (TIF) [file pgen.1002988.s009.tif]

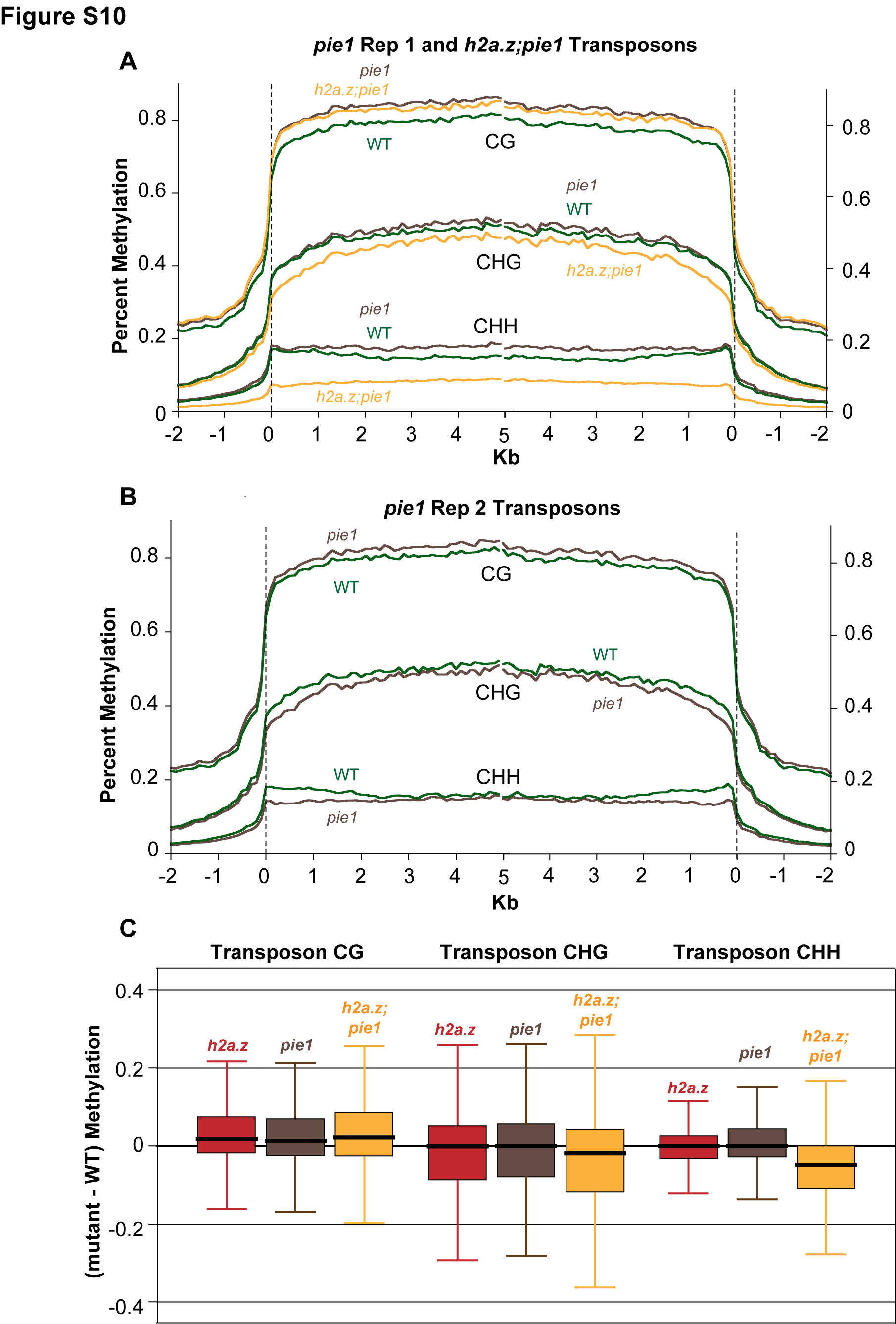

Supplement: Figure S10 — h2a.z;pie1 causes greater loss of transposon methylation than either h2a.z or pie1. (A) Profiles of CG, CHG, and CHH DNA methylation in TEs in seedlings for one replicate each of pie1, h2a.z;pie1 and WT. TEs were aligned as in Figure 3 and average methylation levels for each 100-bp interval are plotted. WT methylation is represented by green traces, pie1 by brown traces, and h2a.z;pie1 by yellow traces. An additional replicate each of pie1 and WT are shown in (B). (C) Box plots of differences in CG, CHG and CHH methylation between WT and either h2a.z, pie1 or h2a.z;pie1 seedlings for all 50 bp windows within TEs. Each box encloses the middle 50% of the distribution, with the horizontal black line marking the median. The lines extending vertically from each box mark the minimum and maximum values that fall within 1.5 times the height of the box. (TIF) [file pgen.1002988.s010.tif]

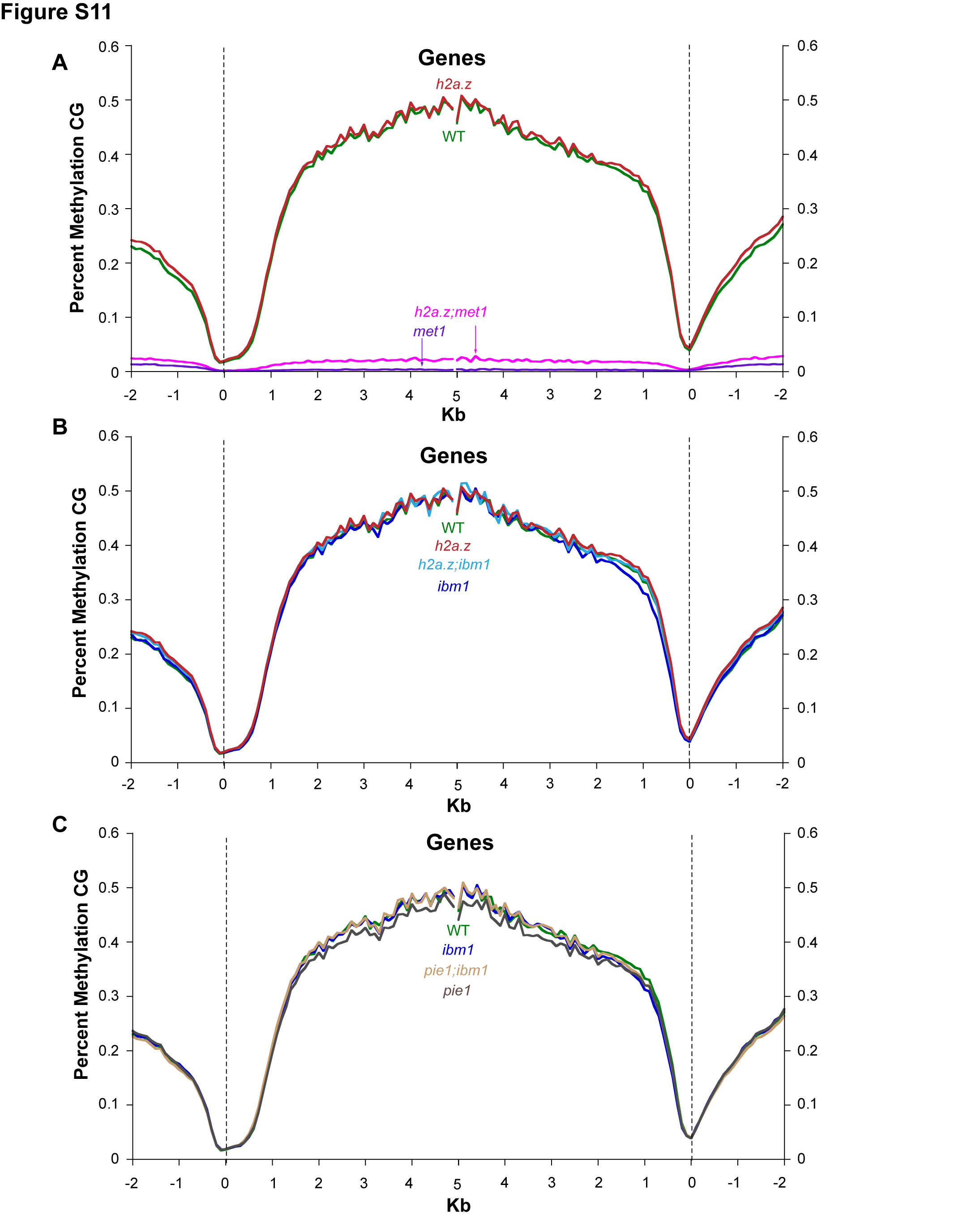

Supplement: Figure S11 — Genic CG DNA methylation profiles of H2A.Z-deficient and DNA methylation-perturbed double mutants. (A) Profiles of CG DNA methylation in h2a.z, h2a.z;met1, met1 and WT. Genes were aligned as in Figure 3 and average methylation levels for each 100-bp interval are plotted from 2 kb away from the gene to 5 kb into the gene. WT methylation is represented by the green trace, h2a.z methylation by the red trace, met1 methylation by the purple trace, and h2a.z;met1 methylation by the pink trace. The dashed line at zero represents the point of alignment. (B) Profiles of CG DNA methylation in genes, as in (A), for h2a.z (red trace), h2a.z;ibm1 (light blue trace), ibm1 (dark blue trace) and WT (green trace). (C) Profiles of CG DNA methylation in genes, as in (A), for pie1 (brown trace), pie1;ibm1 (beige trace), ibm1 (dark blue trace) and WT (green trace). (TIF) [file pgen.1002988.s011.tif]

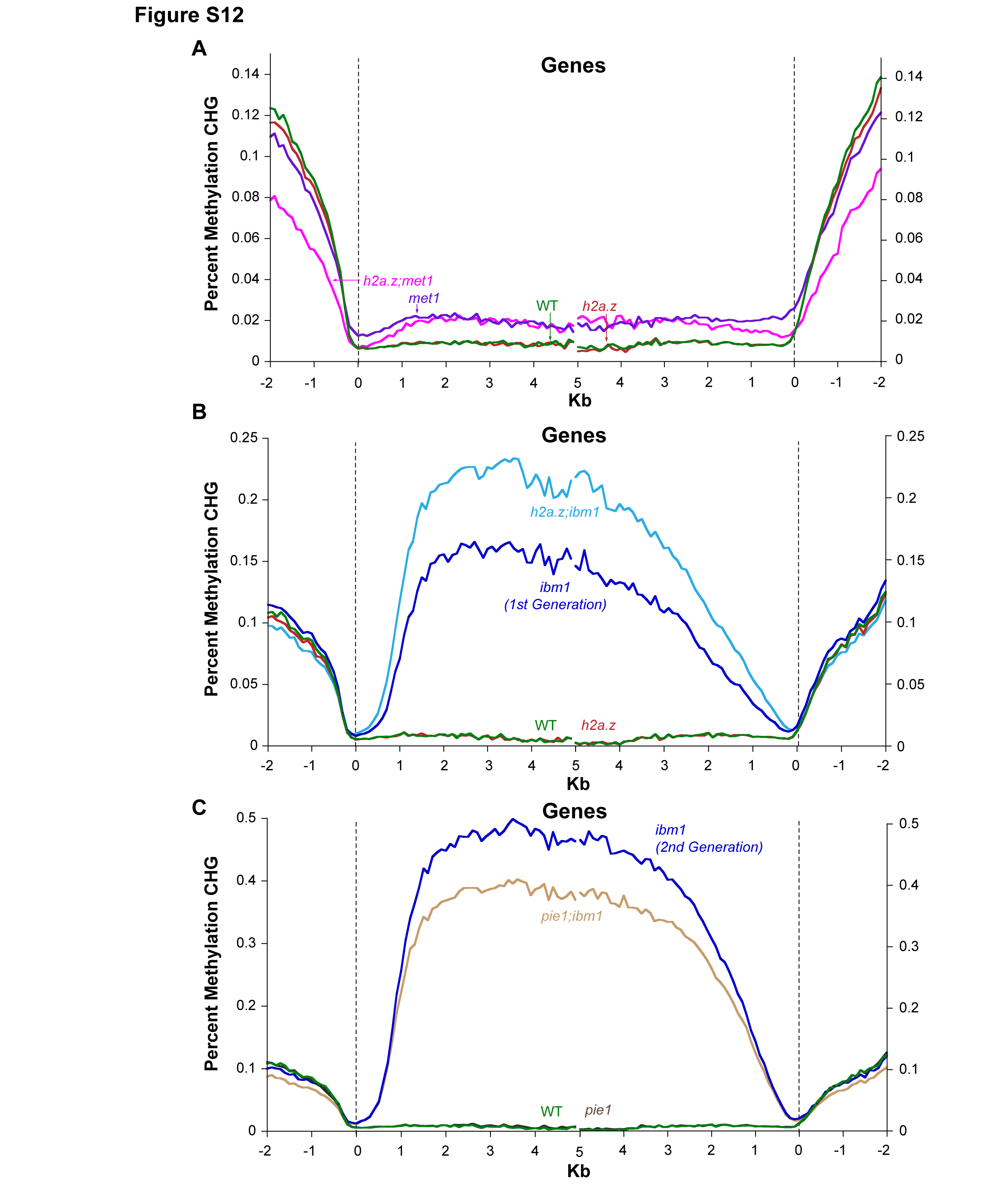

Supplement: Figure S12 — Genic CHG DNA methylation profiles of H2A.Z-deficient and DNA methylation-perturbed double mutants. (A) Profiles of CHG DNA methylation in h2a.z, h2a.z;met1, met1 and WT. Genes were aligned as in Figure 3 and average methylation levels for each 100-bp interval are plotted from 2 kb away from the gene to 5 kb into the gene. WT methylation is represented by the green trace, h2a.z methylation by the red trace, met1 methylation by the purple trace, and h2a.z;met1 methylation by the pink trace. The dashed line at zero represents the point of alignment. (B) Profiles of CHG DNA methylation in genes, as in (A), for h2a.z (red trace), h2a.z;ibm1 (light blue trace), ibm1 (dark blue trace) and WT (green trace). (C) Profiles of CHG DNA methylation in genes, as in (A), for pie1 (brown trace), pie1;ibm1 (beige trace), ibm1 (dark blue trace) and WT (green trace). Note the difference in scale for figures (B) and (C), due to differences in ibm1 CHG hypermethylation between the 1st and 2nd generation plants (discussed in the text). (TIF) [file pgen.1002988.s012.tif]

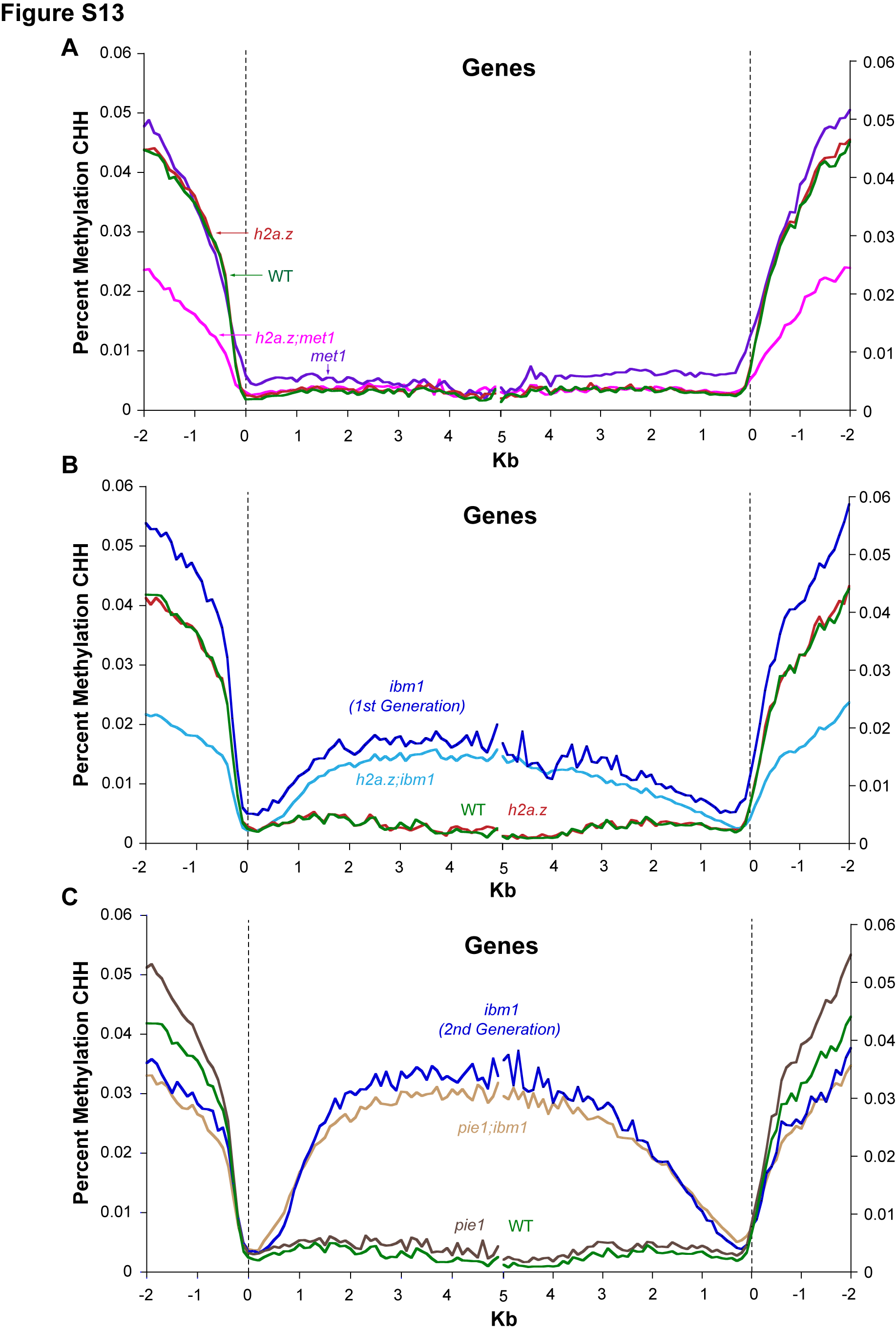

Supplement: Figure S13 — Genic CHH DNA methylation profiles of H2A.Z-deficient and DNA methylation-perturbed double mutants. (A) Profiles of CHH DNA methylation in h2a.z, h2a.z;met1, met1 and WT. Genes were aligned as in Figure 3 and average methylation levels for each 100-bp interval are plotted from 2 kb away from the gene to 5 kb into the gene. WT methylation is represented by the green trace, h2a.z methylation by the red trace, met1 methylation by the purple trace, and h2a.z;met1 methylation by the pink trace. The dashed line at zero represents the point of alignment. (B) Profiles of CHH DNA methylation in genes, as in (A), for h2a.z (red trace), h2a.z;ibm1 (light blue trace), ibm1 (dark blue trace) and WT (green trace). (C) Profiles of CHH DNA methylation in genes, as in (A), for pie1 (brown trace), pie1;ibm1 (beige trace), ibm1 (dark blue trace) and WT (green trace). (TIF) [file pgen.1002988.s013.tif]

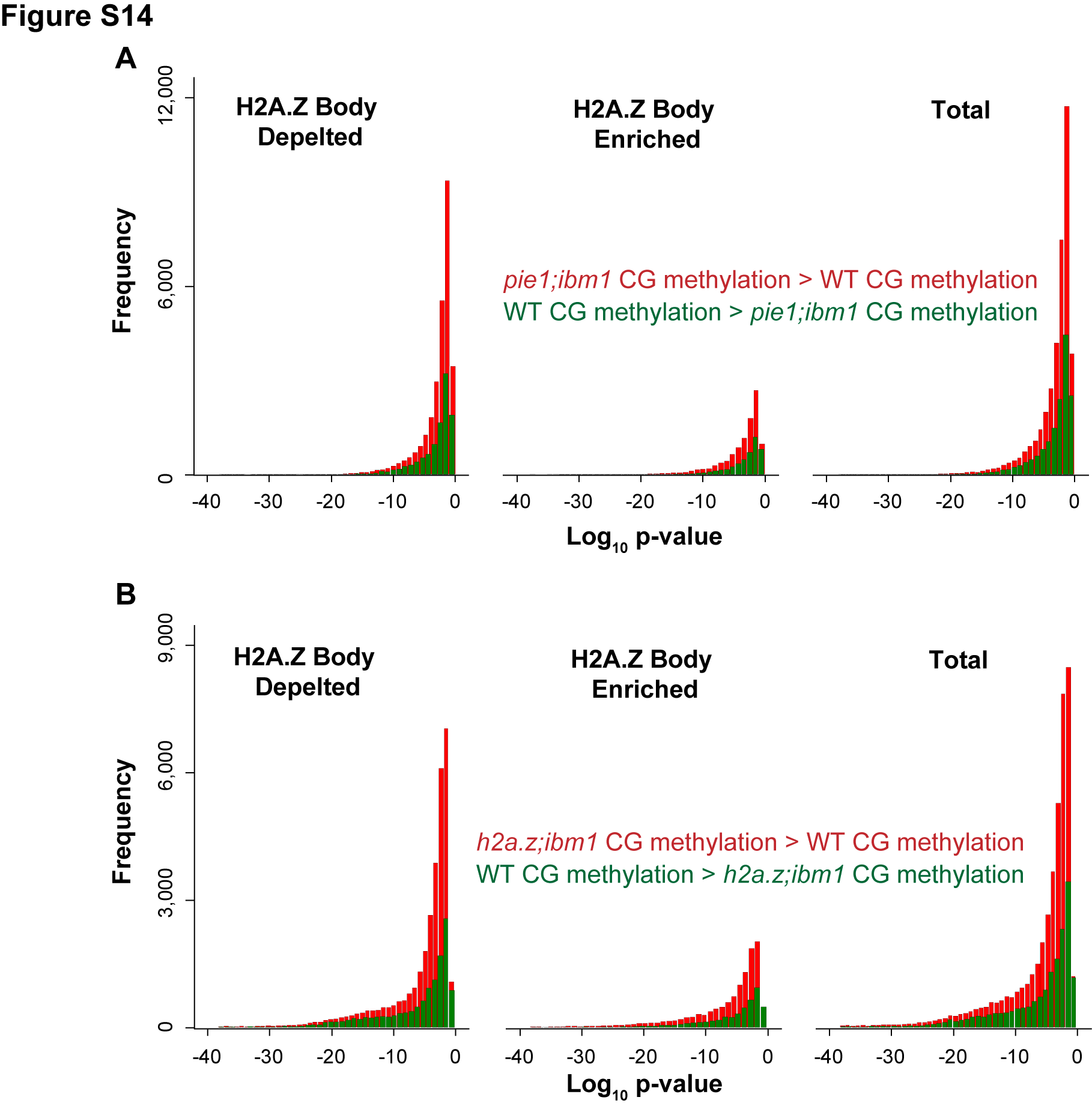

Supplement: Figure S14 — Methylation differences between double mutants and control lines. Frequency plots of the occurrence of 50 bp windows with differential methylation between double mutant and parental control lines in genes, plotted by their p-value significance as in Figure S7. Separate plots are shown for the windows in genes with low H2A.Z in gene bodies, genes with high H2A.Z in gene bodies, and the combined total. Frequency counts for windows with greater methylation in the double mutant than the control are shown in red, while counts for windows with greater methylation in the control than in the double mutant are shown in green. (TIF) [file pgen.1002988.s014.tif]

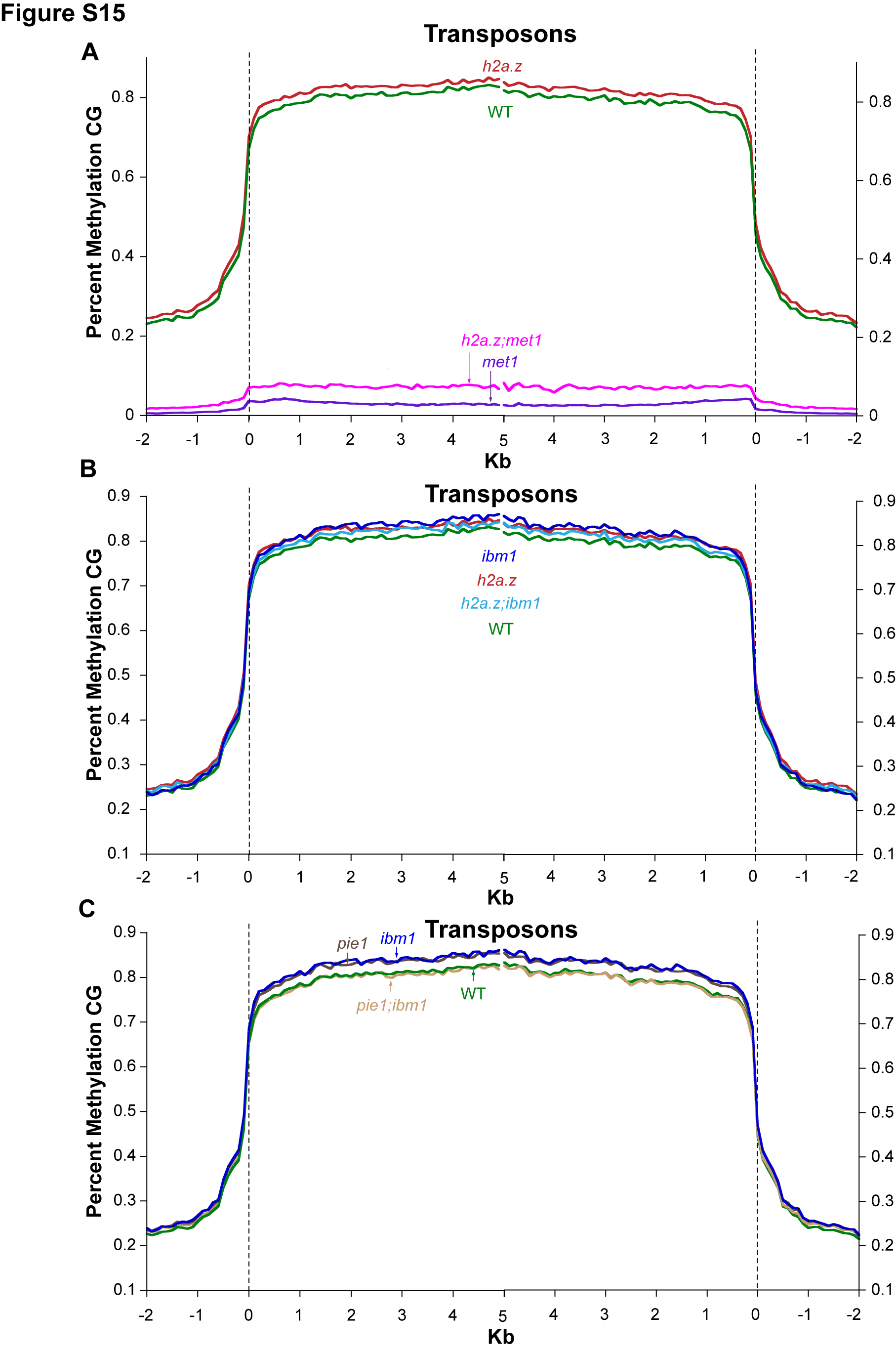

Supplement: Figure S15 — Transposon CG DNA methylation profiles of H2A.Z-deficient and DNA methylation-perturbed double mutants. (A) Profiles of CG DNA methylation in h2a.z;met1, h2a.z, met1 and WT. Transposons were aligned as in Figure 3 and average methylation levels for each 100-bp interval are plotted from 2 kb away from the TE to 5 kb into the TE. WT methylation is represented by the green trace, h2a.z methylation by the red trace, met1 methylation by the purple trace, and h2a.z;met1 methylation by the pink trace. The dashed line at zero represents the point of alignment. (B) Profiles of CG DNA methylation in TEs, as in (A), for h2a.z (red trace), h2a.z;ibm1 (light blue trace), ibm1 (dark blue trace) and WT (green trace). (C) Profiles of CG DNA methylation in TEs, as in (A), for pie1 (brown trace), pie1;ibm1 (beige trace), ibm1 (dark blue trace) and WT (green trace). (TIF) [file pgen.1002988.s015.tif]

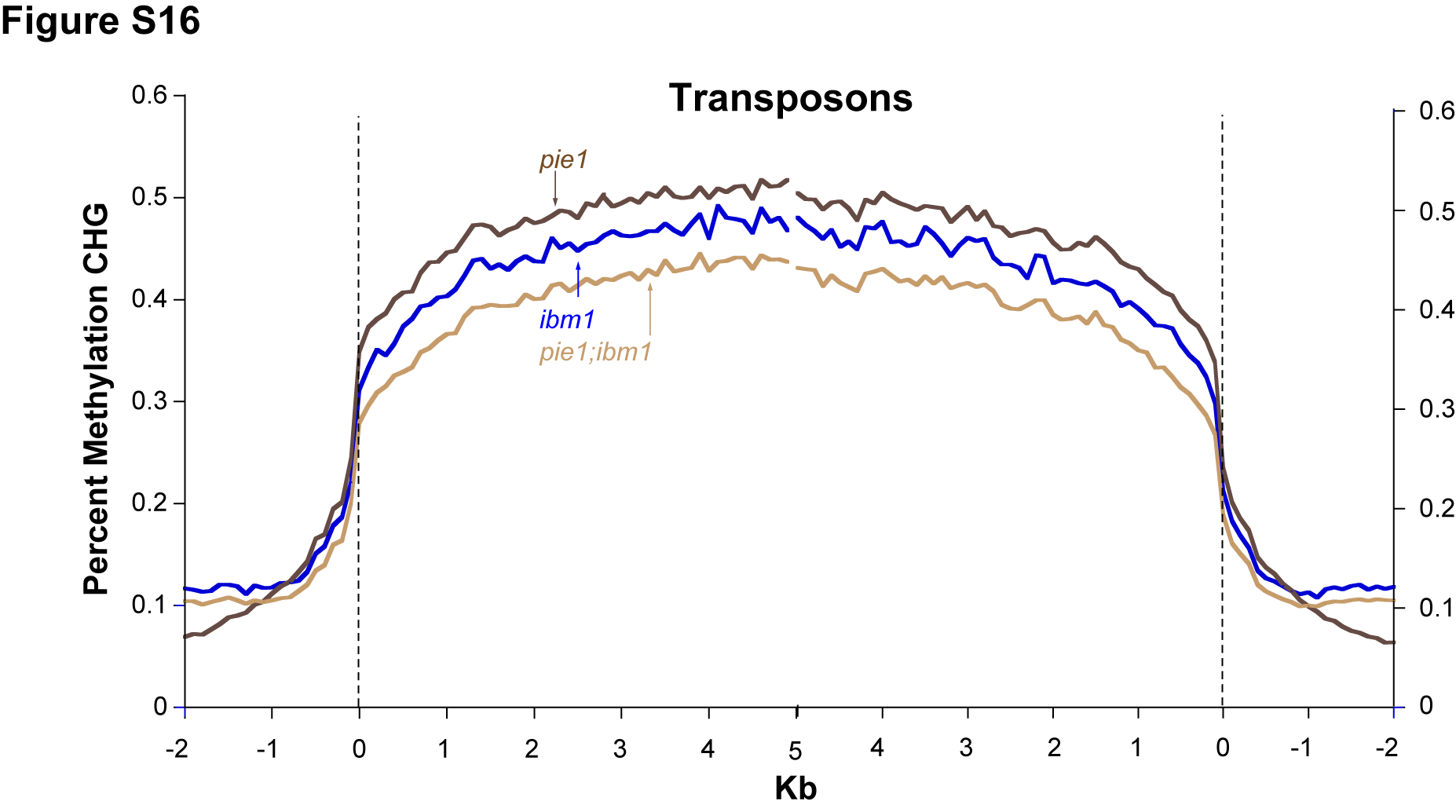

Supplement: Figure S16 — CHG hypomethylation of transposons is greater in pie1;ibm1 than in parental lines. (A) Profiles of CHG DNA methylation in pie1 (brown trace), pie1;ibm1 (beige trace), and ibm1 (dark blue trace). Transposons were aligned as in Figure 3 and average methylation levels for each 100-bp interval are plotted from 2 kb away from the TE to 5 kb into the TE. The dashed line at zero represents the point of alignment. (TIF) [file pgen.1002988.s016.tif]

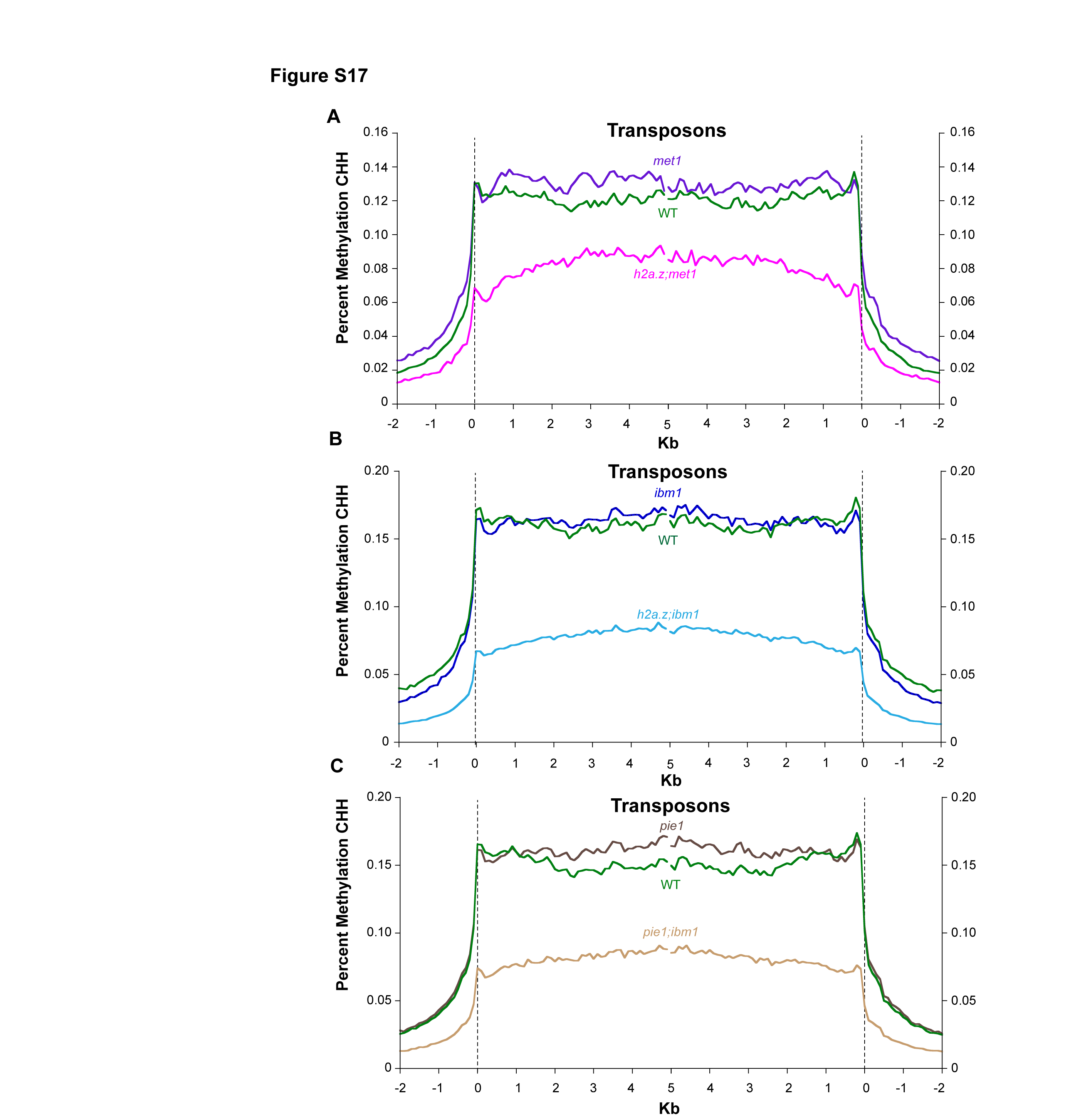

Supplement: Figure S17 — H2A.Z-deficient and DNA methylation-perturbed double mutants show greater loss of TE CHH methylation. (A) Profiles of CHH DNA methylation in h2a.z;met1, met1 and WT. Transposons were aligned as in Figure 3 and average methylation levels for each 100-bp interval are plotted from 2 kb away from the TE to 5 kb into the TE. WT methylation is represented by the green trace, met1 methylation by the purple trace, and h2a.z;met1 methylation by the pink trace. The dashed line at zero represents the point of alignment. (B) Profiles of CHH DNA methylation in TEs, as in (A) for h2a.z;ibm1 (light blue trace), ibm1 (dark blue trace) and WT (green trace). (C) Profiles of CHH DNA methylation in TEs, as in (A), for pie1 (brown trace), pie1;ibm1 (beige trace), and WT (green trace). (TIF) [file pgen.1002988.s017.tif]

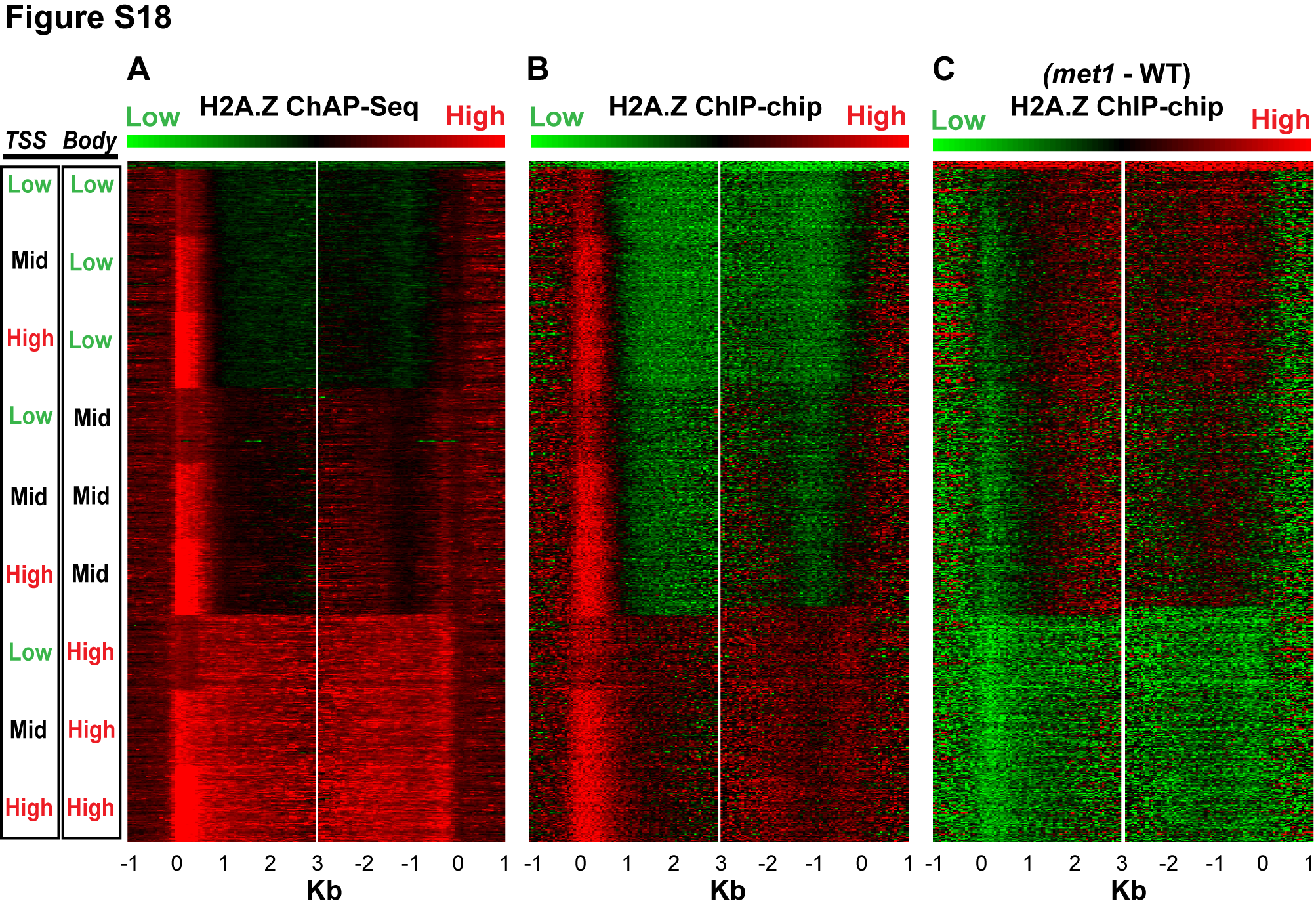

Supplement: Figure S18 — Comparisons of H2A.Z ChAP Sequencing and ChIP-chip data. Heat maps of H2A.Z enrichment across genes in various datasets. (A) Our current H2A.Z ChAP sequencing data, presented exactly as in Figure 4C (for reference). The data were clustered into 9 approximately equal sized groups based on three tiers (low, mid, high) of H2A.Z enrichment at the TSS and across the body (see Figure 4C). (B) Our previous H2A.Z ChIP-chip data from [16], presented using the same clustering as in (A). (C) Our previous ChIP-chip data for H2A.Z enrichment in met1 [16], presented using the same clustering as in (A), shown here as (met1 – WT) to aid in visualization of changes that occur in the met1 mutant. (TIF) [file pgen.1002988.s018.tif]

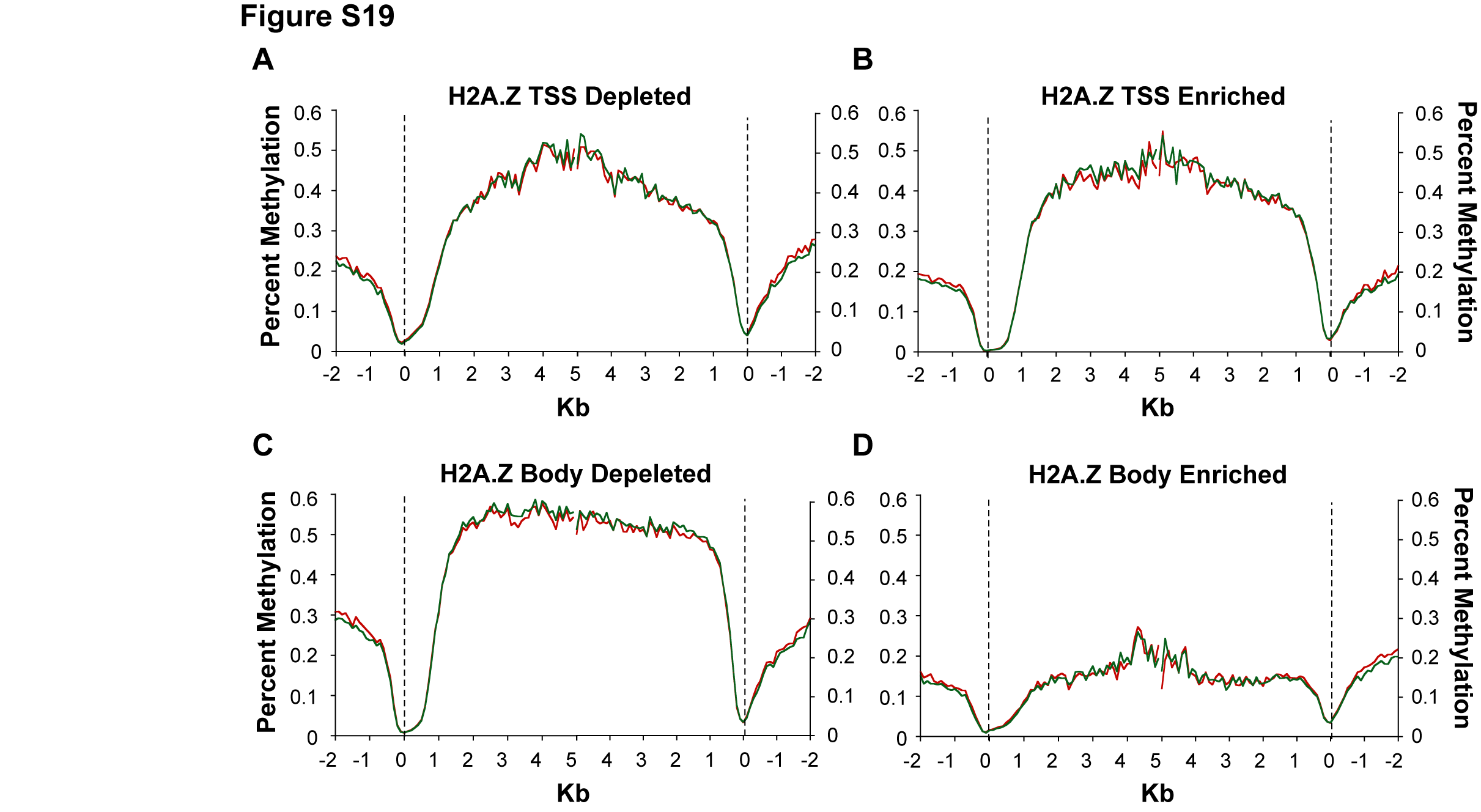

Supplement: Figure S19 — DNA methylation profiles of h2a.z and WT grouped by H2A.Z enrichment. Profiles of CG DNA methylation in the h2a.z and WT datasets, for (A) genes with low H2A.Z at the TSS (n = 3,916), (B) genes with high H2A.Z at the TSS (n = 4,086), (C) genes with low H2A.Z across the gene body (n = 3,920), and (D) genes with high H2A.Z across the gene body (n = 4,081). Genes were aligned as in Figure 3. WT methylation is represented by the green traces, while h2a.z methylation is represented by red traces. The dashed lines at zero represents the point of alignment. (TIF) [file pgen.1002988.s019.tif]

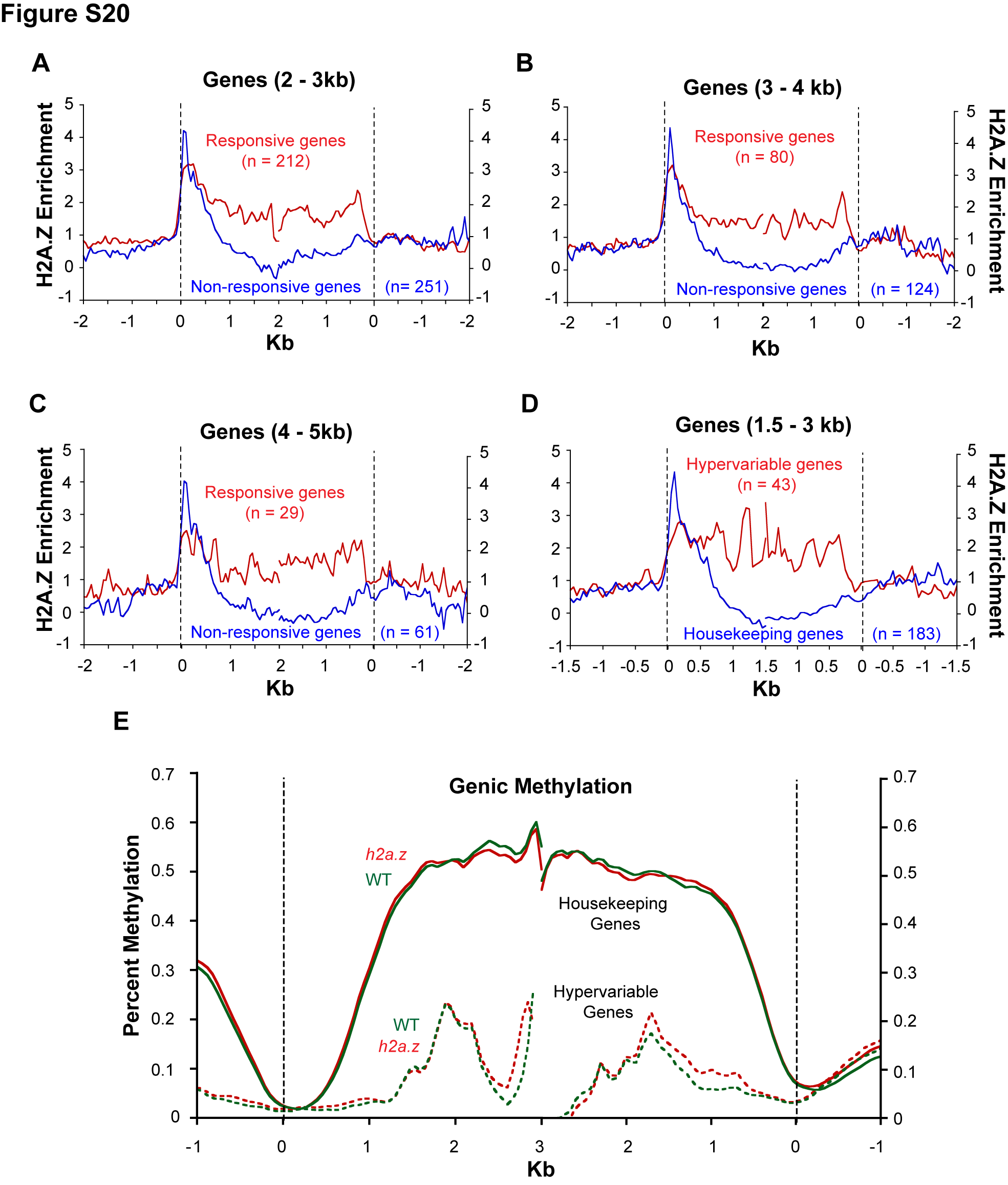

Supplement: Figure S20 — H2A.Z enrichment and methylation in hypervariable and housekeeping genes. (A–C) Average profiles of H2A.Z enrichment (IP - input) across gene bodies. Genes were aligned at the 5′ end (left half of panel) and the 3′ end (right half of panel) and average H2A.Z enrichment levels for each 50-bp interval are plotted from 2 kb away from the gene (negative numbers) to 2 kb into the gene (positive numbers). To avoid averaging H2A.Z enrichment at the 5′ and 3′ ends into the H2A.Z body distribution, we do not use data within 1 kb of the opposite end of the gene. Plots show H2A.Z enrichment in the 1000 most responsive genes as defined in [70], and 1000 randomly selected genes with responsiveness score of 0 (least responsive genes) grouped by length: 2–3 kb in (A), 3–4 kb in (B) and 4–5 kb in (C). The dashed lines at zero represent the points of alignment. (D) Average profiles of H2A.Z enrichment in hypervariable genes and housekeeping genes, as defined in [70], between 1.5 and 3 kb in length. Genes were aligned as in (A–C), except that the alignment was extended 1.5 kb into the gene. (E) DNA methylation in hypervariable and housekeeping genes. Genes were aligned at the 5′ end (left half of panel) and the 3′ end (right half of panel) and average methylation levels for each 100-bp interval are plotted from 1 kb away from the gene (negative numbers) to 3 kb into the gene (positive numbers). (TIF) [file pgen.1002988.s020.tif]

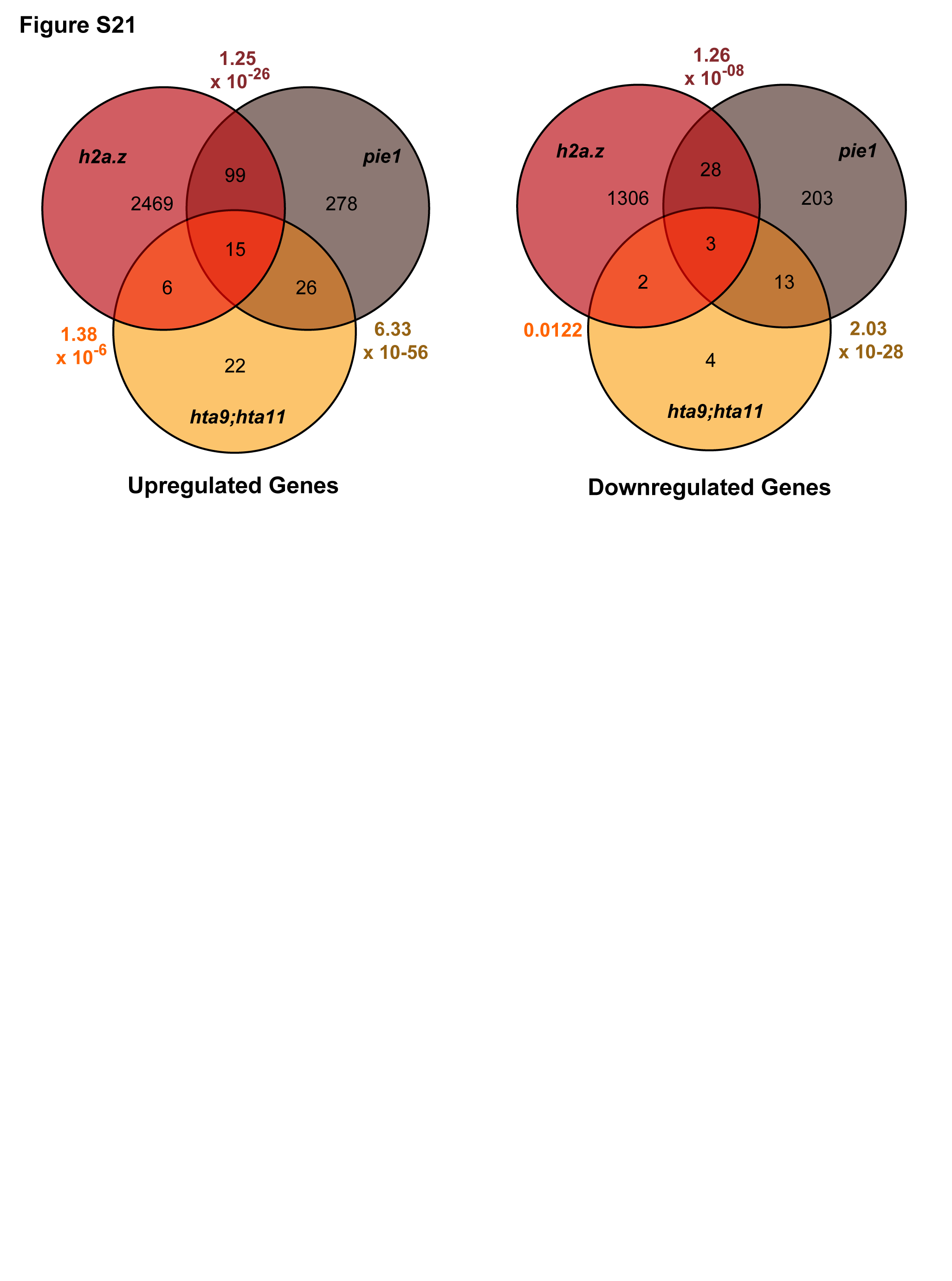

Supplement: Figure S21 — h2a.z, pie1, and hta9;hta11 transcriptional misregulation. Venn diagrams for genes upregulated (A) or downregulated (B) in h2a.z, pie1, and hta9;hta11. The h2a.z data are taken from the RNA sequencing presented here, while both the pie1 and hta9;hta11 data are from previously published lists of misregulated genes in these mutants [37]. Genes are defined here as upregulated if the Log2 (mutant/WT) score is >0.5 (upregulated) or <−0.5 (downregulated). P-values for the associated overlaps between each pair of mutants, calculated using a modified Fisher's Exact test, are indicated outside the Venn diagram where each pair of circles meet. (TIF) [file pgen.1002988.s021.tif]
